# The inner-shell ionization and fragmentation of selenophene at 120 eV

**DOI:** 10.1038/s41598-026-39246-4

**Published:** 2026-02-17

**Authors:** Tiffany Walmsley, Felix Allum, James R. Harries, Yoshiaki Kumagai, Joseph W. McManus, Kiyonobu Nagaya, Mathew Britton, Mark Brouard, Philip H. Bucksbaum, Mizuho Fushitani, Ian Gabalski, Tatsuo Gejo, Paul Hockett, Andrew J. Howard, Hiroshi Iwayama, Edwin Kukk, Chow-shing Lam, Russell S. Minns, Akinobu Niozu, Sekito Nishimuro, Johannes Niskanen, Shigeki Owada, Weronika O. Razmus, Daniel Rolles, James D. Somper, Kiyoshi Ueda, James Unwin, Shin-ichi Wada, Joanne L. Woodhouse, Ruaridh Forbes, Michael Burt, Emily M. Warne

**Affiliations:** 1 Central Laser Facility, STFC Rutherford Appleton Laboratory, Didcot, Oxfordshire, OX11 0QX UK; 2 Chemistry Research Laboratory, Department of Chemistry, University of Oxford, Oxford, OX1 3TA UK; 3 Linac Coherent Light Source, SLAC National Accelerator Laboratory, 2575 Sand Hill Road, Menlo Park, 94025 CA USA; 4 PULSE Institute, SLAC National Accelerator Laboratory, 2575 Sand Hill Road, Menlo Park, 94025 CA USA; 5 Deutsches Elektronen-Synchrotron, DESY, Notkestr. 85, 22607 Hamburg, Germany; 6 National Institutes for Quantum Science and Technology (QST), SPring-8, 1-1-1 Kouto, Sayo, Hyogo, 679-5148 Japan; 7 Department of Applied Physics, Tokyo University of Agriculture and Technology, Tokyo, Japan; 8 Department of Physics, Nara Women’s University, Nara, 630-8506 Japan; 9 Department of Physics, Kyoto University, Kyoto, 606-8502 Japan; 10 Department of Chemistry, Graduate School of Science, Nagoya University, Nagoya, Aichi, 464-8602 Japan; 11 Department of Applied Physics, Stanford University, Stanford, 94305-4090 CA USA; 12 Graduate School of Science, University of Hyogo, Kouto 3-2-1, Kamigori-cho, Ako-gun, Hyogo, 678-1297 Japan; 13 National Research Council of Canada, 100 Sussex Dr., Ottawa, K1A 0R6 ON Canada; 14 Institute for Molecular Science, Okazaki, 444-8585 Japan; 15 Department of Physics and Astronomy, University of Turku, FI-20014 Turku, Finland; 16 School of Chemistry and Chemical Engineering, University of Southampton, Highfield, Southampton, SO17 1BJ UK; 17 Graduate School of Humanities and Social Sciences, Hiroshima University, Higashi-Hiroshima, 739-8524 Japan; 18 Department of Materials Sciences and Engineering, School of Materials and Chemical Technology, Institute of Science Tokyo, 2-12-1 W4-10 Ookayama, Meguro-ku, Tokyo, 152-8550 Japan; 19 Japan Synchrotron Radiation Research Institute, Kouto 1-1-1 Sayo, Hyogo, Japan; 20 RIKEN SPring-8 Centre, Kouto 1-1-1 Sayo, Hyogo, Japan; 21 J. R. Macdonald Laboratory, Department of Physics, Kansas State University, Manhattan, 66506 KS USA; 22 Department of Chemistry, Tohoku University, Sendai, 980-8578 Japan; 23 Graduate School of Advanced Science and Engineering, Hiroshima University, Higashi-Hiroshima, 739-8526 Japan; 24 Department of Chemistry, University of California, Davis, One Shields Avenue, Davis, 95616 CA USA; 25 Department of Chemistry, Trent University, 1600 West Bank Drive, Peterborough, K9L 0G2 ON Canada

**Keywords:** Chemistry, Physics

## Abstract

The inner-shell ionization of selenophene at 120 eV produces a rich array of fragmentation dynamics, including many originating from Auger-Meitner processes. In this report, three-dimensional velocity-map imaging and covariance analysis were used to identify and characterize over 50 distinct selenophene fragmentation channels. The majority resulted in two or three ‘heavy’ products containing selenium or carbon, many of which had identical mass-to-charge ratios but different chemical compositions due to the degree of hydrogenation and the selenium isotope involved. Covariance analysis was used to isolate these reaction channels and to provide estimates of their relative yields. In combination with prior similar studies on thiophene and furan, the current results indicate that the nature of the heteroatom significantly influences the charge redistribution and bond cleavage dynamics induced by the Auger-Meitner process, and demonstrate the sensitivity of inner-shell ionization dynamics to the molecular and electronic structures of heterocyclic systems.

## Introduction

Inner-shell spectroscopy, induced by intense X-ray or extreme ultraviolet (XUV) light, enables the nuclear and electronic structures of few-atom molecules to be probed comprehensively, often with quantum state-specific detail^1–5^. In these measurements, the initial ionization event can lead to Auger-Meitner decay, whereby the atomic core orbital vacancy created by the incident radiation is filled by a higher-lying electron^6^. The accompanying energy release results in further electron loss and the creation of a multiply-charged molecular ion^7,8^. The fragmentation pathways of such ions can provide information about their parent structures, particularly in the high-charge regimes used for Coulomb explosion imaging mass spectrometry (CEI-MS), where the fragment trajectories reflect the initial bonding structure of the molecule^9–12^. Furthermore, since the initial core excitation produces a photoelectron, correlated ion-electron measurements can match particular photoions to specific quantum states^1,2^. When inner-shell ionization and CEI-MS are coupled with laser pump-probe schemes, they can be used to follow the evolving electronic and nuclear configurations of a molecule over time, allowing for ‘real-time’ reaction imaging^3,5^.

Despite the significant potential of combining inner-shell ionization and CEI-MS, many fundamental questions remain about the experimental methodology, particularly with respect to distinguishing how molecules fragment following their initial ionization. The Auger-Meitner decay of a molecular species can proceed through an enormous number of similar pathways. These can differ by the initial charge locations on the molecular ion, the relative times at which these charged sites are formed, and how the charge is redistributed as the molecule relaxes^13–16^. These factors also dictate whether the photoion remains intact or fragments, potentially through a variety of competing pathways that produce virtually identical products (e.g., hydrocarbons that differ by a single hydrogen atom).

To investigate these questions, the present study considers how the fragmentation dynamics of aromatic heterocycles are influenced by the nature of the heteroatom involved in the initial core ionization. In particular, we examine the breakup of selenophene ($\hbox {C}_4\hbox {H}_4\hbox {Se}$) following site-selective ionization of the selenium 3d orbital, and compare the results to previously reported measurements involving thiophene ($\hbox {C}_4\hbox {H}_4\hbox {S}$) and furan ($\hbox {C}_4\hbox {H}_4\hbox {O}$)^17–20^. These species are of broad interest due to their roles in diverse chemical and biological processes. For example, selenophene and its derivatives are used in several pharmaceutical, electrochemical, and optoelectronic applications^21–24^; thiophene is found in various semiconductors^25^; and furan is a building block of many natural products^26^. Pertinent to this work, the polycationic states of these heterocycles exhibit a range of dynamics, including outcomes involving many-body fragmentation, isomerization, ring-opening, and hydrogen loss or migration^17–20^. They are, therefore, excellent candidates for studying how different heteroatoms influence molecular fragmentation dynamics following core ionization.

In the experiments reported here, site-selective probing of selenophene was carried out via selenium 3d ionization using 120 eV photons from the Spring-8 Angstrom Compact Free Electron Laser (SACLA)^27^. This photon energy primarily induces valence electron Auger-Meitner (MVV) decay and hence a broad distribution of initial charge sites. Three-dimensional velocity map imaging (VMI) mass spectrometry and photoion-photoion covariance analysis were then used to distinguish and characterize the resulting fragmentation dynamics of the molecular ions^28–34^. The mass resolution of the spectrometer was sufficiently precise to separate fragments that differed by as little as one atomic mass unit, which enabled product channels involving different selenium isotopes and numbers of hydrogen atoms to be assigned^16^. The principal fragmentation pathways involved the cleavage of both carbon-selenium bonds, or one carbon-carbon bond and one carbon-selenium bond, with varying degrees of secondary hydrocarbon dissociation and hydrogen loss, as well as evidence of hydrogen migration. These outcomes are similar to, but have markedly different branching ratios than, those exhibited by thiophene and furan, suggesting that the nature of the heteroatom greatly influences the overall distribution of reaction outcomes.

## Results

### Product ion mass spectrum and covariance

**Fig. 1:**
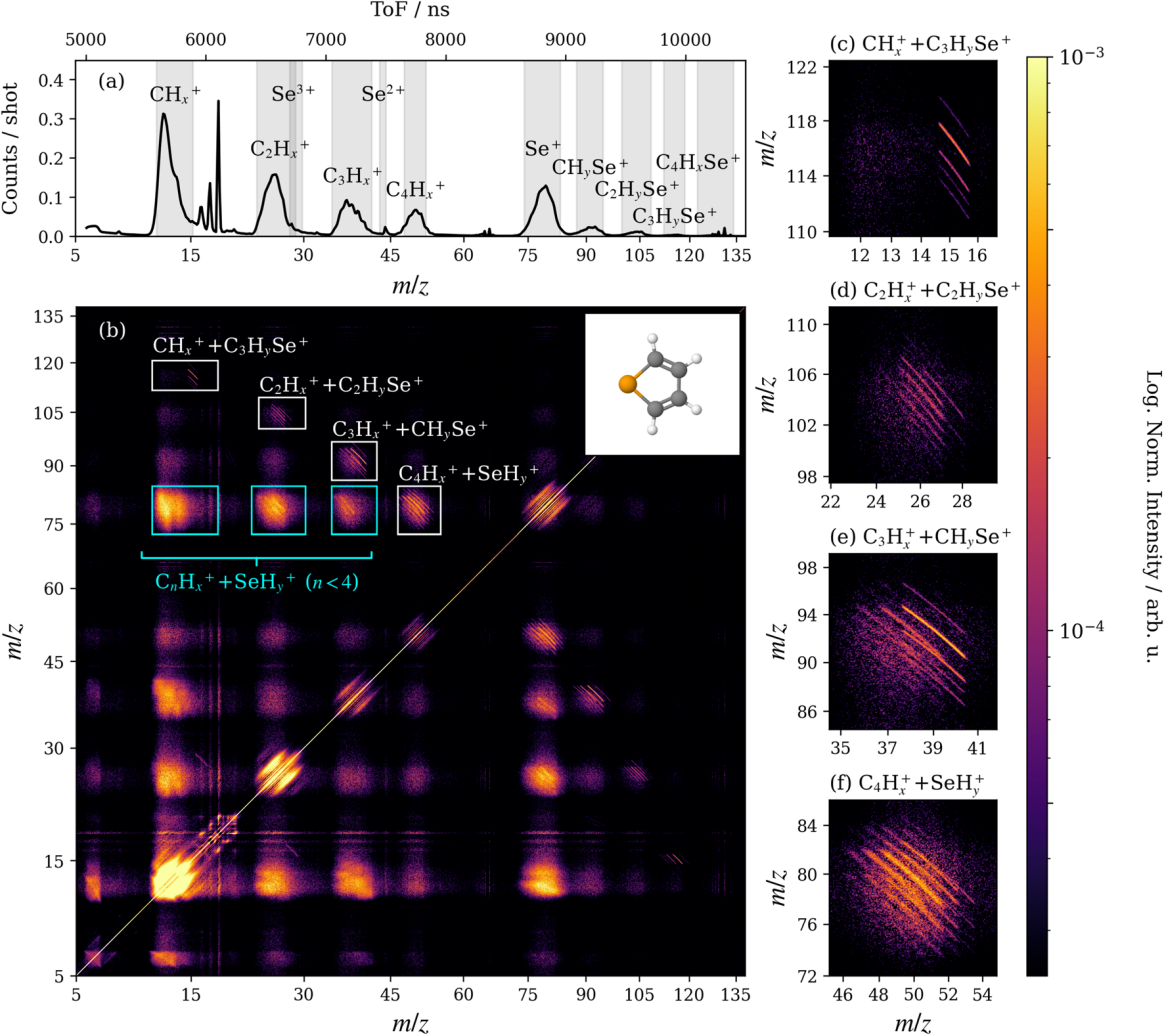
(**a**) The mass-to-charge (*m*/*z*) spectrum of selenophene following ionization at 120 eV. The grey regions highlight the ranges of ions that contain the same numbers of carbon and selenium atoms. (**b**) Covariance mapping was used to identify pairs of correlated ion species. The covariance map was calculated using ten contingent covariance subsets, as described in the Methods section. The geometry of the neutral parent molecule is given in the top right. Note the nonlinear *m*/*z* axes, which are plotted linearly in time-of-flight, and that the minimum *m*/*z* shown is five, as no meaningful covariance was seen below this limit due to scattered light signals. Features corresponding to the major groups of two- and many-body fragmentation channels are indicated by white and cyan boxes, respectively. The regions enclosed by the white boxes are given in greater detail in panels (**c-f**).

The mass spectrum generated following the ionization of selenophene at 120 eV is given in Figure 1(a). This was recorded under single-photon conditions, which were established prior to the selenophene measurements by using the photoionization of iodine 4d electrons in methyl iodide as a reference. These conditions were then verified in selenophene by determining that the relative intensities of the covariances calculated across the contingent covariance bins did not vary as a function of probe pulse energy. The principal products, highlighted by the grey-shaded regions, are $\hbox {Se}^+$ and ions of the form $\hbox {C}_n\hbox {H}_x^{+}$ and $\hbox {C}_n\hbox {H}_x\hbox {Se}^{+}$, with $n=1-4$ and $x\le 4$. Selenium cations were also observed in their +2 and +3 charge states (the latter overlapping with $\hbox {C}_2\hbox {H}_x^+$), but with much lower intensities than $\hbox {Se}^+$. These peaks are generally broad due to variations in the number of hydrogen atoms as well as the presence of different selenium isotopes, of which ^76^Se, ^77^Se, ^78^Se, ^80^Se and ^82^Se are present in significant quantities (i.e., greater than 1%; the natural isotope abundances are provided in Table 1). For instance, it can be shown using covariance analysis that the $\hbox {Se}^{+}$ distribution also contains contributions from $\hbox {SeH}^{+}$ and $\hbox {SeH}_{2}^{+}$. We also note that, although many of the above fragments imply the loss of neutral or ionic hydrogen, the operating conditions of the detector prevented $\hbox {H}^{+}$ from being reliably measured (see the Methods section for further details). Even so, the presence or absence of these species can often be inferred from the momenta of the other detected fragments, as will be shown below.

Time-of-flight covariance mapping, shown in Figure 1(b), reveals which pairs of ions can be produced from the same fragmentation channels^30,31^. Intense and well-defined positive covariances are seen for most combinations of $\hbox {C}_n\hbox {H}_x^{+}$ and $\hbox {C}_{(4-n)}\hbox {H}_y\hbox {Se}^{+}$, where $x+y\le 4$, as well as for most $\hbox {C}_n\hbox {H}_x^{+}$ and $\hbox {Se}^+$/$\hbox {SeH}_y^+$ pairs, where $y\le 2$. The former, which are indicated by white boxes in Figure 1(b) and expanded in greater detail in panels (c)-(f), exhibit linear features with gradients of approximately −1 (the slight curvature exhibited in (c)-(f) is a well-understood VMI field effect)^35^. These are characteristic of products generated with anti-correlated momenta following two-body fragmentation, which is consistent with the parent molecule breaking in two; either by cleaving both C-Se bonds, one C-Se and one C-C bond, or two C-C bonds^36^. It should be emphasized, however, that the loss of one or more hydrogens (either neutral or ionic) would impart very little momentum to the heavier $\hbox {C}_n\hbox {H}_x^{+}$ and $\hbox {C}_{(4-n)}\hbox {H}_y\hbox {Se}^{+}$ fragments, meaning that it is not possible to exclude the possibility of further hydrogen loss in these channels. For ease of discussion, the term two-body fragmentation is used hereon to refer to fragmentation pathways that generate $\hbox {C}_n\hbox {H}_x^{+}$ and $\hbox {C}_{(4-n)}\hbox {H}_y\hbox {Se}^{+}$ fragments, but we note that these processes can still be accompanied by varying degrees of $\hbox {H}^{0/+}$ loss.

The covariances observed between the $\hbox {C}_n\hbox {H}_x^{+}$ fragments where $n<4$ and $\hbox {Se}^+$/$\hbox {SeH}_y^+$ (the cyan boxes in Figure 1) are attributed to many-body fragmentation pathways that generate at least two carbon-containing products. For example, the correlation between $\hbox {C}_3\hbox {H}_x^+$ and $\hbox {Se}^+$ indicates that these fragments are accompanied by a second carbon species, which can be neutral or positively charged. Unlike the well-resolved $\hbox {C}_n\hbox {H}_x^{+}$ and $\hbox {C}_{(4-n)}\hbox {H}_y\hbox {Se}^{+}$ covariance features, the ionic products generated from many-body fragmentation processes do not typically recoil at $180^{\circ }$ from each other. Their signatures therefore overlap to create the diffuse features observed in the covariance map^36^. These contributions are difficult to unequivocally resolve across all pairs of ions, but can in some cases be assigned to sequential many-body fragmentation mechanisms occurring via an initial charge separation step into two heavy cations (i.e., those containing carbon or selenium), followed by secondary dissociation of at least one of these fragments^36^. As in the previous case, these processes can again involve additional $\hbox {H}^{0/+}$ loss. Further details on these assignments are given in the following sections.

### Processes generating $\hbox {C}_n\hbox {H}_x^{+}$ and $\hbox {C}_{(4-n)}\hbox {H}_y\hbox {Se}^{+}$

#### Isolating overlapping channels

**Fig. 2:**
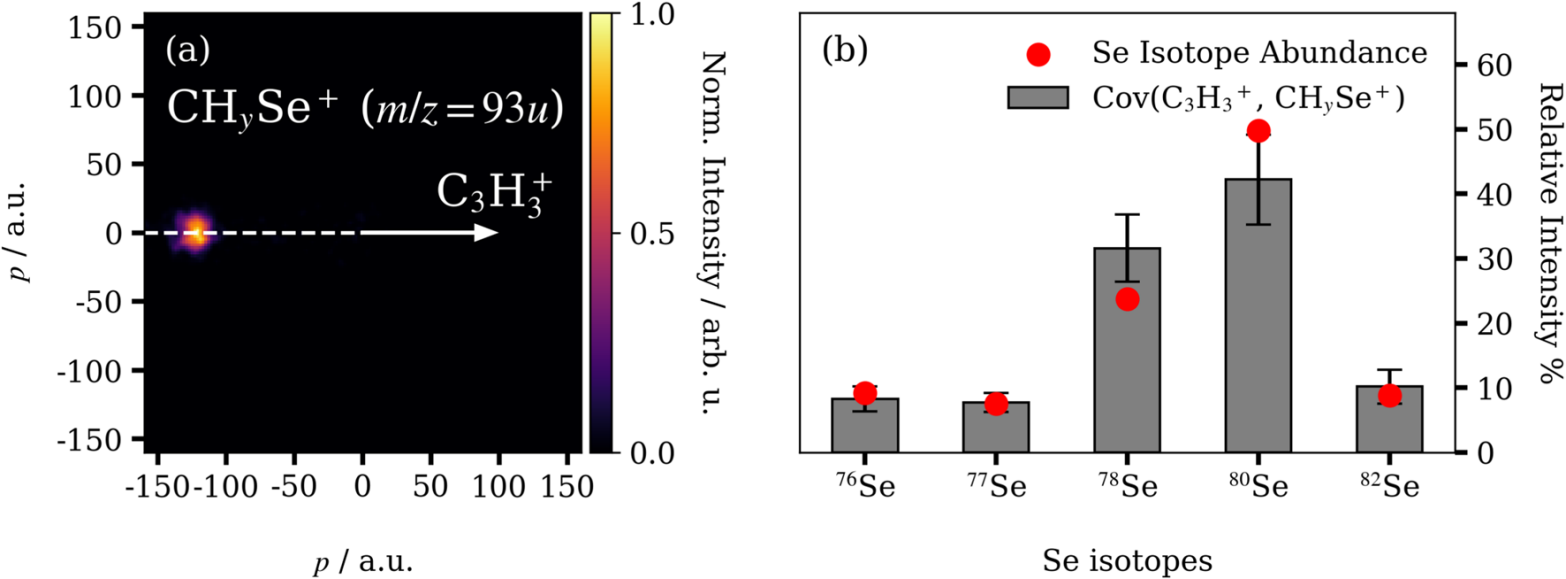
(**a**) The recoil-frame covariance map of the fragment momentum distribution of $\hbox {CH}_y\hbox {Se}^+$ (*m*/*z* = 93 *u*) with respect to $\hbox {C}_3\hbox {H}_3^+$. The momenta (*p*) are given in atomic units (a.u.). (**b**) The summed relative intensities of fragmentation channels producing $\hbox {C}_3\hbox {H}_3^+$ and all $\hbox {CH}_y\hbox {Se}^+$ co-fragments incorporating the different selenium isotopes, as described in Table 1. The error bars correspond to uncertainty values obtained from an adapted bootstrapping method outlined in the Methods section. Red dots indicate the natural selenium isotope abundances.

The covariant $\hbox {C}_n\hbox {H}_x^{+}$ and $\hbox {C}_{(4-n)}\hbox {H}_y\hbox {Se}^{+}$ features observed in Figure 1(b) arise from overlapping fragmentation channels that differ by the selenium isotope and the number of hydrogen atoms involved (e.g., $\hbox {CH}^{76}\hbox {Se}^{+}$ and $\hbox {C}^{77}\hbox {Se}^{+}$ have the same nominal mass). These can be further distinguished by covariance analysis of the three-dimensional fragment ion momenta, as described in the Methods section. Like time-of-flight covariance mapping, three-dimensional covariance imaging identifies pairs of ions that are generated together, but has the additional advantage that reaction channels can be further characterized for a given ion pair by their relative fragment momenta. By way of example, the recoil-frame covariance map of $\hbox {CH}_{{y}}\hbox {Se}^{+}$ relative to $\hbox {C}_3\hbox {H}_3^{+}$ is shown in Figure 2(a). The recoil vector of $\hbox {C}_3\hbox {H}_3^{+}$, the reference ion, is given by the white arrow, and the observed signal in the map indicates the fragment momenta of covariant $\hbox {CH}_{{y}}\hbox {Se}^{+}$ products. Here, the covariance map exhibits a single intense $\hbox {CH}_{{y}}\hbox {Se}^{+}$ feature that is recoiling at $180^{\circ }$ from $\hbox {C}_3\hbox {H}_3^{+}$, which suggests the parent ion is breaking into two ionic fragments. In this case, only $\hbox {CH}_{{y}}\hbox {Se}^{+}$ ions with a total mass of 93 *u* were used in the covariance analysis, and the degree of hydrogenation of the reference ion $\hbox {C}_3\hbox {H}_3^{+}$ is known, so the product channels that can contribute to this feature must be limited to the following two cases:

1 $$\begin{aligned} & \text {C}_4\text {H}_4{}^{80}\text {Se}^{2+}\rightarrow \text {C}_3\text {H}_3^+ + \text {CH}^{80}\text {Se}^{+}, \end{aligned}$$

2 $$\begin{aligned} & \text {C}_4\text {H}_4{}^{80}\text {Se}^{2/3+}\rightarrow \text {C}_3\text {H}_3^+ + \,^{13}\text {C}^{80}\text {Se}^{+} + \text {H}^{0/+}. \end{aligned}$$

Of these, the second channel is far less likely due to the low isotopic abundance (1.1%) of carbon-13. As such, contributions from this isotope are not considered further.

Using the above procedure, recoil-frame covariance images were produced for all possible pairs of $\hbox {C}_3\hbox {H}_3^+$ and $\hbox {CH}_y\hbox {Se}^+$ (with $m/z=$) 88-95). As in the previous example, these exhibited contributions from only two possible fragmentation channels:

3 $$\begin{aligned} & \text {C}_4\text {H}_4\text {Se}^{2+}\rightarrow \text {C}_3\text {H}_3^+ + \text {CHSe}^{+}, \end{aligned}$$

4 $$\begin{aligned} & \text {C}_4\text {H}_4\text {Se}^{2/3+}\rightarrow \text {C}_3\text {H}_3^+ + \text {CSe}^{+} + \text {H}^{0/+}. \end{aligned}$$

The above outcomes suggest that, for these ion pairs, doubly- or triply-charged selenophene ions are formed prior to fragmentation. This is consistent with the charge distribution exhibited by krypton, which is electronically similar to selenium, following single-photon absorption at 120 eV^37^. At this photon energy, krypton is ionized to its +2 and +3 charge states in a 2.5:1 ratio, with a much smaller fraction of +1 states.

The magnitude of the covariance measured between a pair of ions is proportional to their populations, assuming that all fragmentation channels are detected with uniform efficiency^30^. As such, to determine the relative yields of the fragmentation channels listed above, the integrated intensities of the Newton-frame covariance features for each $\hbox {C}_3\hbox {H}_3^+$/$\hbox {CH}_y\hbox {Se}^+$ pair were extracted and partitioned according to the natural abundances of the selenium isotopes involved. For example, the magnitude of the $\hbox {CH}_y\hbox {Se}^+$ (*m*/*z* = 89) covariance, which can only come from channels producing $\hbox {CH}^{76}\hbox {Se}^+$ or $\hbox {C}^{77}\hbox {Se}^+$ (ignoring carbon-13), was measured to be 2.8 ± 0.7 in arbitrary units (arb. u.). The selenium-76 and −77 isotopes have relative abundances of 9.2% and 7.6%, respectively, meaning that they should proportionally contribute 1.7 ± 0.5 and 1.4 ± 0.4 arb. u. of intensity to the observed feature (i.e., the selenium-76 isotope should be 21% more abundant than selenium-77). This logic rests on the assumption that all possible fragmentation channels that can occur do occur, and is borne out by the measured data, as will be detailed below.

The integrated intensities for each $\hbox {C}_3\hbox {H}_3^+$/$\hbox {CH}_y\hbox {Se}^+$ pair are given in Table 1. As discussed above, in each case only a limited number of selenium isotopes contribute to these intensities, and they are assumed to exist in their natural abundances. The contributions of each isotope are separated into different columns in Table 1, and their sums provide an overall measure of the relative abundances of the selenium isotopes involved in the product channels. Figure 2(b) demonstrates that these values are in excellent agreement with the naturally observed isotope abundances, suggesting that all possible fragmentation channels described by reactions (3) and (4) occurred, as assumed previously. The slight deviations between the expected and extracted isotope abundances are likely due to one or more channels being over- or underestimated, or due to overlapping channels that could not be completely isolated using the procedures outlined in the Methods section. Even so, we note that these discrepancies are small for this set of ion pairs.

In a similar manner to the $\hbox {C}_3\hbox {H}_3^+$/$\hbox {CH}_y\hbox {Se}^+$ example, in-depth covariance analyses were carried out for all of the features highlighted by the white boxes in Figure 1(b), including $\hbox {C}_4\hbox {H}_x^+$/$\hbox {SeH}_y^+$, the remaining $\hbox {C}_3\hbox {H}_x^+$/$\hbox {CH}_y\hbox {Se}^+$ configurations, $\hbox {C}_2\hbox {H}_x^+$/$\hbox {C}_2\hbox {H}_y\hbox {Se}^+$, and $\hbox {CH}_x^+$/$\hbox {C}_3\hbox {H}_y\hbox {Se}^+$. These are collected in Section 1 of the Supplementary Information (Figures S1 to S18) and broadly support the conclusion that most fragments originate from doubly- or triply-charged selenophenes through channels that are analogous to reactions (3) and (4). These can be expressed more generally as:

5 $$\begin{aligned} & \text {C}_4\text {H}_4\text {Se}^{2+}\rightarrow \text {C}_n\text {H}_x^+ + \text {C}_{(4-n)}\text {H}_{(4-x)}\text {Se}^{+}, \end{aligned}$$

6 $$\begin{aligned} & \text {C}_4\text {H}_4\text {Se}^{2/3+}\rightarrow \text {C}_n\text {H}_x^+ + \text {C}_{(4-n)}\text {H}_{y}\text {Se}^{+} + (4-x-y)\text {H}^{0/+}. \end{aligned}$$

**Table 1:** The relative integrated covariance intensities (in arbitrary units) of correlated $\hbox {C}_3\hbox {H}_3^+$/$\hbox {CH}_y\hbox {Se}^+$ ($m/z=88-95$) pairs, partitioned according to their contributing selenium isotopes. The summed intensities obtained for each isotope (bottom row) are also compared with their natural abundances (top row).

$\hbox {CH}_y\hbox {Se}^+$ *m*/*z*	Intensity (arb. u.)	^76^Se (9.2%)	^77^Se (7.6%)	^78^Se (23.7%)	^80^Se (49.8%)	^82^Se (8.8%)
88	0.5 ± 0.3	0.5 ± 0.2				
89	2.8 ± 0.7	1.5 ± 0.4	1.3 ± 0.3			
90	2.6 ± 0.7		0.6 ± 0.2	2.0 ± 0.5		
91	5.7 ± 1.1			5.7 ± 1.1		
92	1.4 ± 0.6				1.4 ± 0.6	
93	8.9 ± 1.6				8.9 ± 1.6	
94	0.6 ± 0.3					0.6 ± 0.3
95	1.9 ± 0.5					1.9 ± 0.5
	$\Sigma$Intensity (%)	8.3 ± 1.9	7.8 ± 1.5	31.6 ± 5.2	42.2 ± 7.0	10.2 ± 2.6

#### Relative fragmentation channel intensities

The entries in the isotope columns of Table 1 represent unique fragmentation channels. For example, the lone item in the $\hbox {CH}_y\hbox {Se}^+$ ($m/z=$ 93) row can be assigned to reaction (1). Using this information, as well as the additional fragmentation data provided in Tables S1-S18 of the Supplementary Information, the relative intensities of each distinguishable $\hbox {C}_n\hbox {H}_x^{+}$/$\hbox {C}_{(4-n)}\hbox {H}_y\hbox {Se}^{+}$ fragmentation pathway were determined. These are summarized in Table 2, which lists all channels with relative intensities that are greater than 1% of the total (22 less prominent channels are additionally included in Table S19, but together constitute only 12.3% of the aggregate). It should be emphasized that only two-body fragmentations where all hydrogen atoms are accounted for, such as those described by reaction (5), could be definitively assigned. These are labeled (i) to (vii) and all originate from selenophene dications (an eighth dication channel producing $\hbox {CH}_3^+$ and $\hbox {C}_3\hbox {HSe}^+$ is also listed in Table S19). The most abundant of these produced $\hbox {C}_4\hbox {H}_3^+$ with $\hbox {SeH}^+$ (channel (ii), 6.1 ± 0.6%) and $\hbox {C}_3\hbox {H}_3^+$ with $\hbox {CHSe}^+$ (channel (v), 6.3 ± 0.7%). The remaining channels listed in Table 2 all exhibit some degree of hydrogen loss; however, the experiment was not able to distinguish whether these were neutral or ionic, so it is not possible to unequivocally determine the identity of the parent charge states in these cases. That being said, single-photon absorption at 120 eV should primarily create selenophene di- or trications in a roughly 2.5:1 ratio, as noted above^37^. As such, in the absence of multi-photon effects, it is unlikely that these hydrogen loss channels released more than one proton.

Two clear trends emerge from the data in Table 2: first, channels involving the cleavage of two C-Se bonds are the most abundant, which likely reflects the weakness of the C-Se bonds relative to the C-C bonds^38,39^; and second, channels involving hydrogen loss are much more common than those where they remain bound to the selenophene fragments, possibly due to the excess energy available following Auger-Meitner decay. More details on potential $\hbox {H}^{0/+}$ dynamics occurring within these channels are provided in Section 3 of the Supplementary Information.

**Fig. 3:**
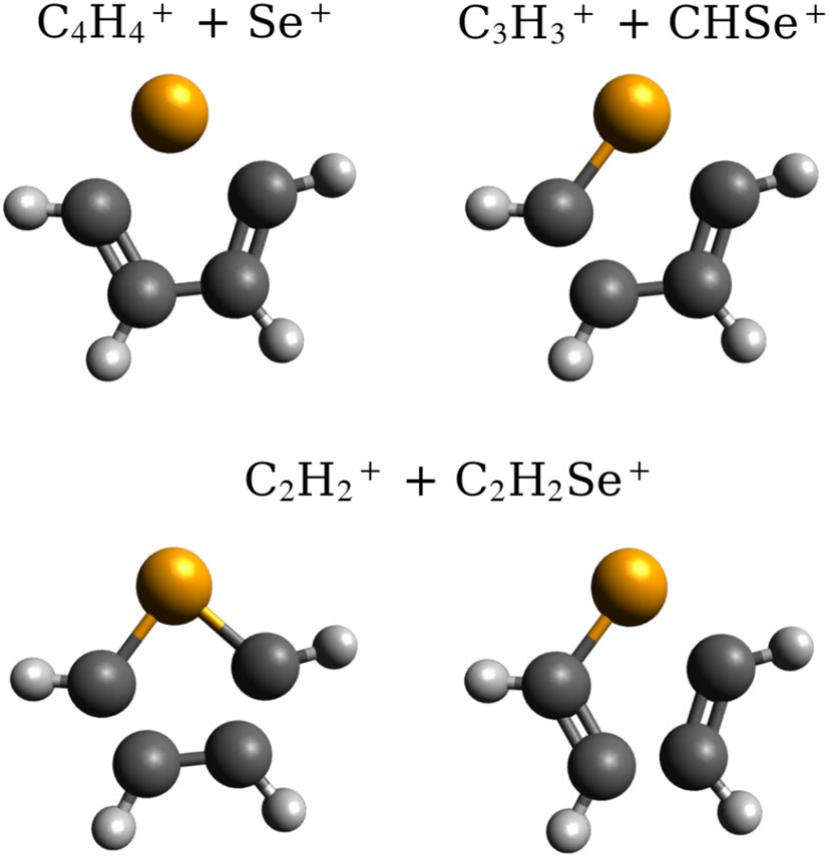
Selenophene illustrations showing the C-Se and/or C-C bonds required to break to form the products of two-body fragmentation channels (iii), (v), and (vi) in Table 2.

Over half of the $\hbox {C}_n\hbox {H}_x^{+}$/$\hbox {C}_{(4-n)}\hbox {H}_y\hbox {Se}^{+}$ fragmentation pathways (54.0 ± 1.7%) involve the breakage of both C-Se bonds, producing $\hbox {C}_4\hbox {H}_x^+$ and $\hbox {SeH}_y^+$, as illustrated for channel (iii) in Figure 3. Among these, the dominant channel formed $\hbox {C}_4\hbox {H}_2^+$, $\hbox {Se}^+$, and two $\hbox {H}^{0/+}$, with a relative intensity of 7.9 ± 0.7%. However, as previously stated, it was not possible to determine whether the hydrogens were released as neutral atoms, protons, or as a molecule. Overall, $\hbox {C}_4\hbox {H}_x^+$ and $\hbox {SeH}_y^+$ fragments are 2.7 times more likely to be produced through many-body hydrogen loss channels that are similar to reaction (6), rather than through the two-body fragmentations defined by reaction (5).

All of the other observed $\hbox {C}_n\hbox {H}_x^{+}$/$\hbox {C}_{(4-n)}\hbox {H}_y\hbox {Se}^{+}$ channels require two C-C bonds to break or cleave one C-Se bond and a C-C bond, as depicted in Figure 3 for channels (v) and (vi). As shown, the products of channel (vi), along with many others listed in Table 2, can be formed via different combinations of C-Se and C-C bond cleavage. Fragmentation channels yielding $\hbox {C}_3\hbox {H}_x^+$ and $\hbox {CH}_y\hbox {Se}^+$ co-products were the second most abundant group, making up 23.2 ± 1.1% of the total. Within this group, the most intense pathway was the $\hbox {C}_3\hbox {H}_3^+$ and $\hbox {CHSe}^+$ product pair detailed in Figure 2 and Table 1, which had a relative abundance of 6.3 ± 0.7%. These pathways are 1.5 times more likely to involve hydrogen loss than not. Channels that yielded $\hbox {C}_2\hbox {H}_x^+$ and $\hbox {C}_2\hbox {H}_y\hbox {Se}^+$ co-products represent 15.7 ± 0.8% of the total. In this case, the most prominent pathway formed $\hbox {C}_2\hbox {H}_2^+$, $\hbox {C}_2\hbox {HSe}^+$, and $\hbox {H}^{0/+}$, and had a relative intensity of 3.1 ± 0.4%. For these channels, the hydrogen loss pathways were 3.5 times more likely to occur than the purely two-body fragmentation routes. Finally, the only significant $\hbox {CH}_x^+$/$\hbox {C}_3\hbox {H}_y\hbox {Se}^+$ channel involved the migration of two hydrogen atoms to form $\hbox {CH}_3^+$, $\hbox {C}_3\hbox {Se}^+$, and $\hbox {H}^{0/+}$. This occurred with an intensity of 4.5 ± 0.6%.

**Table 2:** The relative integrated intensities of ‘two-body’ fragmentation channels (excluding channels with relative intensities <1%, which sum to 12.3%) obtained via recoil-frame covariance and isotope abundance considerations. All channels, including those with relative intensities <1%, are also presented in Table S19 in the SI. Uncertainties were determined using an adapted bootstrapping method.

Ion Pair	Fragmentation Channel	Rel. Intensity (%)	$\Sigma$ Intensity (%)
$\hbox {C}_4\hbox {H}_x^+$ + $\hbox {SeH}_y^+$	$\hbox {C}_4^+$ + $\hbox {Se}^+$ + 4$\hbox {H}^{0/+}$	1.6$\,\pm \,$0.3	53.0$\,\pm \,$1.7
	$\hbox {C}_4^+$ + $\hbox {SeH}^+$ + 3$\hbox {H}^{0/+}$	2.4$\,\pm \,$0.4	
	$\hbox {C}_4\hbox {H}^+$ + $\hbox {Se}^+$ + 3$\hbox {H}^{0/+}$	4.5$\,\pm \,$0.4	
	$\hbox {C}_4\hbox {H}^+$ + $\hbox {SeH}^+$ + 2$\hbox {H}^{0/+}$	5.3$\,\pm \,$0.6	
	$\hbox {C}_4\hbox {H}^+$ + $\hbox {SeH}_2^+$ + $\hbox {H}^{0/+}$	3.0$\,\pm \,$0.3	
	$\hbox {C}_4\hbox {H}_2^+$ + $\hbox {Se}^+$ + 2$\hbox {H}^{0/+}$	7.9$\,\pm \,$0.7	
	$\hbox {C}_4\hbox {H}_2^+$ + $\hbox {SeH}^+$ + $\hbox {H}^{0/+}$	7.9$\,\pm \,$0.6	
	(i) $\hbox {C}_4 \hbox {H}_2^+$ + $\hbox {SeH}_2^+$	5.6$\,\pm \,$0.5	
	$\hbox {C}_4 \hbox {H}_3^+$ + $\hbox {Se}^+$ + $\hbox {H}^{0/+}$	6.1$\,\pm \,$0.6	
	(ii) $\hbox {C}_4 \hbox {H}_3^+$ + $\hbox {SeH}^+$	6.1$\,\pm \,$0.6	
	(iii) $\hbox {C}_4 \hbox {H}_4^+$ + $\hbox {Se}^+$	2.6$\,\pm \,$0.4	
$\hbox {C}_3 \hbox {H}_x^+$ + $\hbox {CH}_y \hbox {Se}^+$	$\hbox {C}_3^+$ + $\hbox {CH}_3 \hbox {Se}^+$ + $\hbox {H}^{0/+}$	1.1$\,\pm \,$0.3	18.2$\,\pm \,$1.0
	$\hbox {C}_3 \hbox {H}^+$ + $\hbox {CSe}^+$ + 3$\hbox {H}^{0/+}$	1.1$\,\pm \,$0.2	
	$\hbox {C}_3 \hbox {H}^+$ + $\hbox {CHSe}^+$ + 2$\hbox {H}^{0/+}$	1.6$\,\pm \,$0.3	
	(iv) $\hbox {C}_3 \hbox {H}^+$ + $\hbox {CH}_3 \hbox {Se}^+$	1.6$\,\pm \,$0.3	
	$\hbox {C}_3 \hbox {H}_2^+$ + $\hbox {CSe}^+$ + 2$\hbox {H}^{0/+}$	1.3$\,\pm \,$0.3	
	$\hbox {C}_3 \hbox {H}_2^+$ + $\hbox {CHSe}^+$ + $\hbox {H}^{0/+}$	3.4$\,\pm \,$0.4	
	$\hbox {C}_3 \hbox {H}_3^+$ + $\hbox {CSe}^+$ + $\hbox {H}^{0/+}$	1.9$\,\pm \,$0.3	
	(v) $\hbox {C}_3 \hbox {H}_3^+$ + $\hbox {CHSe}^+$	6.3$\,\pm \,$0.7	
$\hbox {C}_2 \hbox {H}_x^+$ + $\hbox {C}_2 \hbox {H}_y \hbox {Se}^+$	$\hbox {C}_2 \hbox {H}^+$ + $\hbox {C}_2 \hbox {Se}^+$ + 3$\hbox {H}^{0/+}$	1.0$\,\pm \,$0.2	11.9$\,\pm \,$0.7
	$\hbox {C}_2 \hbox {H}_2^+$ + $\hbox {C}_2 \hbox {Se}^+$ + 2$\hbox {H}^{0/+}$	2.1$\,\pm \,$0.3	
	$\hbox {C}_2 \hbox {H}_2^+$ + $\hbox {C}_2 \hbox {HSe}^+$ + $\hbox {H}^{0/+}$	3.1$\,\pm \,$0.4	
	(vi) $\hbox {C}_2 \hbox {H}_2^+$ + $\hbox {C}_2 \hbox {H}_2 \hbox {Se}^+$	1.5$\,\pm \,$0.2	
	$\hbox {C}_2 \hbox {H}_3^+$ + $\hbox {C}_2 \hbox {Se}^+$ + $\hbox {H}^{0/+}$	2.6$\,\pm \,$0.4	
	(vii) $\hbox {C}_2 \hbox {H}_3^+$ + $\hbox {C}_2 \hbox {HSe}^+$	1.6$\,\pm \,$0.3	
$\hbox {CH}_x^+$ + $\hbox {C}_3 \hbox {H}_y \hbox {Se}^+$	$\hbox {CH}_3^+$ + $\hbox {C}_3 \hbox {Se}^+$ + $\hbox {H}^{0/+}$	4.5$\,\pm \,$0.6	4.5$\,\pm \,$0.6
	Total	87.7$\,\pm \,$2.2	

### Processes generating $\hbox {C}_n \hbox {H}_x^{+}$, $\hbox {Se}^+$/$\hbox {SeH}_y^+$ and another carbon-containing fragment

Many-body fragmentation channels producing $\hbox {Se}^+$/$\hbox {SeH}_y^+$ and $\hbox {C}_n \hbox {H}_x^{+}$ with at least one other carbon-containing product are highlighted by the cyan boxes in Figure 1(b). The integrated intensities of these features are 3.5 times larger than those yielding $\hbox {C}_n \hbox {H}_x^{+}$ and $\hbox {C}_{(4-n)} \hbox {H}_y \hbox {Se}^{+}$. However, despite their relative abundance, it is significantly more difficult to isolate contributions to these many-body features from individual reaction channels, as their product momenta overlap to a greater degree. Even so, Newton-frame covariance analysis was able to distinguish at least three fragmentation pathways involving  $^{80}\hbox{Se}^+/^{78} \hbox {SeH}_2^+$: two with $\hbox {C}_2 \hbox {H}_2^+$ and one with $\hbox {C}_3 \hbox {H}^+$.

**Fig. 4:**
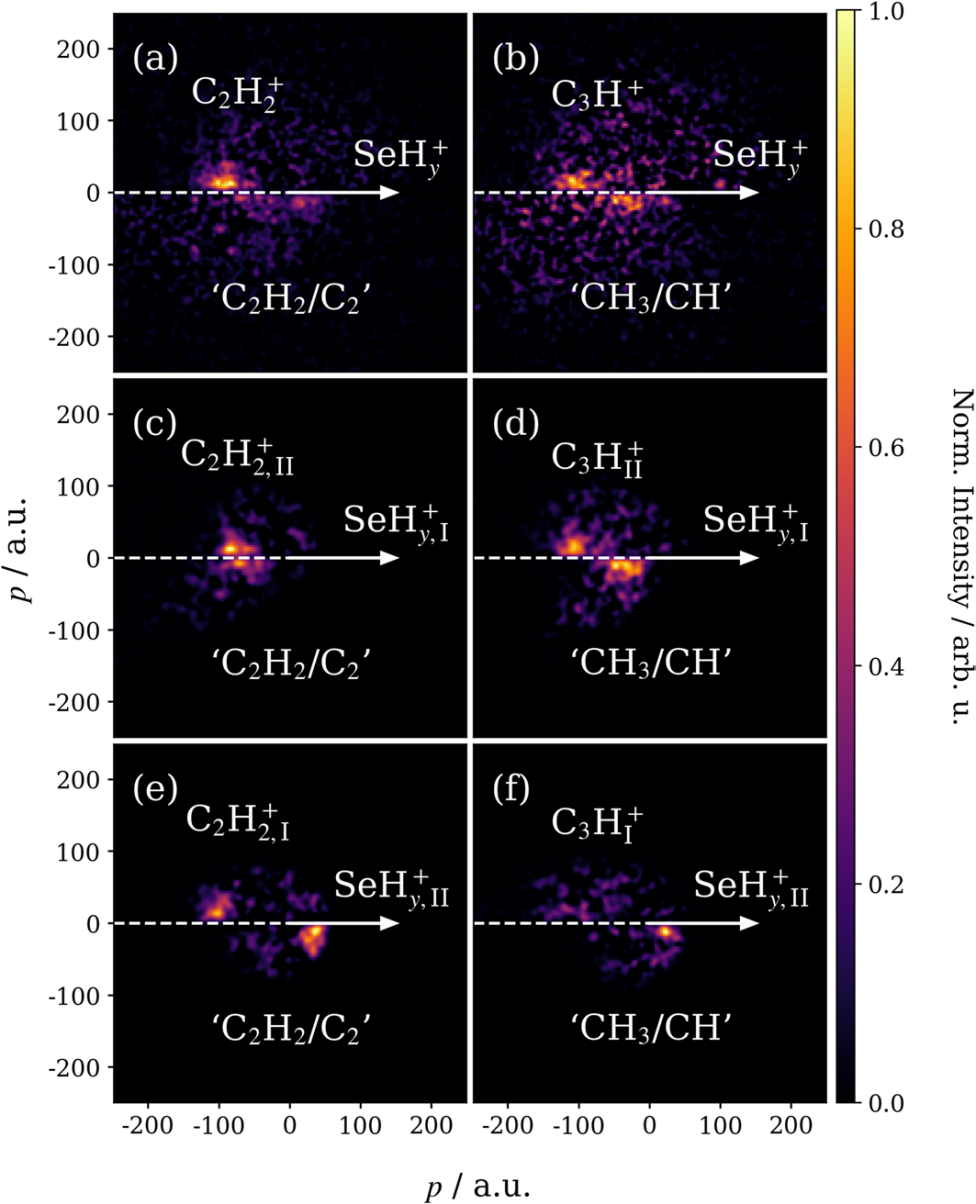
(**a**) and (**b**) are the Newton-frame covariance maps of the momenta of $\hbox {C}_2 \hbox {H}_2^+$ and $\hbox {C}_3 \hbox {H}^+$ relative to $\hbox {SeH}_y^+$ (with $y=$ 0 or 2), calculated without momentum constraints. In each case, the momentum corresponding to the remainder of the molecule (‘$\hbox {C}_2 \hbox {H}_2$/$\hbox {C}_2$’ or ‘$\hbox {CH}_3$/CH’ assuming a three-body fragmentation process) is plotted in the bottom half of the image. Momentum constraints, as described in the main text, were applied to extract the components of the overall distributions that correspond to different primary fragmentation mechanisms; (**c**) and (**d**) are the distributions obtained for mechanisms where $\hbox {SeH}_y^+$ is a primary product ($\hbox {SeH}_{y,\textrm{I}}^+$), while (**e**) and (**f**) are the distributions obtained when it is a secondary product ($\hbox {SeH}_{y,\textrm{II}}^+$). The Newton-frame covariance maps are all normalized to their own maxima, and a Gaussian blur has been added to mitigate the low signal-to-noise ratios of these channels.

Figures 4(a) and (b) illustrate the relative momenta of $\hbox {C}_2 \hbox {H}_2^+$ and $\hbox {C}_3 \hbox {H}^+$, referenced to the recoil of $^{80}\hbox{Se}^+/^{78} \hbox {SeH}_2^+$. In each case, the momentum distribution of the partner ion is plotted in the top half of the figure, while the inferred momentum of the rest of the molecule, which is assumed to remain intact (i.e., ‘$\hbox {C}_2 \hbox {H}_2$’ or ‘$\hbox {CH}_3$’), is plotted in the bottom half. Both perspectives indicate that the two fragments recoil at approximately $180^{\circ }$, which suggests that the remainder of the molecule is neutral (ignoring potential $\hbox {H}^{0/+}$ loss, as discussed previously)^16,40^.

More detailed insight into the fragmentation dynamics can be gained by examining the product momentum distributions. These are considerably broader than those seen for the two-body pathways (e.g., as shown in Figure 2(a) for $\hbox {C}_3 \hbox {H}_3^+$ and $\hbox {CH}_y \hbox {Se}^+$), which suggests contributions from multiple fragmentation mechanisms. In this case, $^{80}\hbox{Se}^+/^{78} \hbox {SeH}_2^+$ can potentially be produced through concerted or sequential reactions as a primary or secondary fragment (denoted hereon with $\textrm{I}$ and $\textrm{II}$ subscripts, respectively). A concerted fragmentation is defined here as one where all bonds are broken within a vibrational period. By contrast, sequential fragmentations proceed through two or more distinct steps involving unstable intermediates. Conservation of momentum arguments, discussed in Section 4 of the Supplementary Information, dictate that $^{80}\hbox{Se}^+/^{78} \hbox {SeH}_2^+$ fragments produced from these pathways will have a broad range of momentum. Sequential reactions that proceed through an initial (primary) charge separation step, as shown in reaction (7) for a triatomic dication $\hbox {ABC}^{2+}$, have particularly distinct fragment momenta from those produced by deferred (secondary) charge separation or concerted mechanisms.

7 $$\begin{aligned} \text {ABC}^{2+}&\rightarrow \text {A}^+ + \text {BC}^{+}\rightarrow \text {A}^+ + \text {B}^+ + \text {C} \end{aligned}$$

In this case, the momentum of the secondary $\hbox {B}^{+}$ product ($\vec {p}_\text {B}$) can be expressed as a function of the fragment masses ($m_{\text {B}}$, $m_{\text {BC}}$) and the momentum gained from the primary and secondary fragmentation events ($\vec {p}_{\text {I}}$, $\vec {p}_{\text {II}}$)^36^:

8 $$\begin{aligned} \vec {p}_\text {B} = -\frac{m_{\text {B}} \vec {p}_{\text {I}}}{m_{\text {BC}}} + \vec {p}_{\text {II}}. \end{aligned}$$

Constraining the Newton-frame covariance calculations to only consider ions that satisfy equation (8) therefore allows fragmentation mechanisms that proceed through an initial charge separation step to be isolated. Figures 4(c) and (d) illustrate the result for $^{80}\hbox{Se}^+_\textrm{I}/^{78} \hbox {SeH}_{2,\textrm{I}}^+$, while (e) and (f) show the same for $^{80}\hbox {Se}^+_{\textrm{II}}/^{78} \hbox {SeH}_{2,\textrm{II}}^+$. Comparing these results to the unconstrained covariance analyses in (a) and (b) confirms that $^{80}\hbox {Se}^+/^{78} \hbox {SeH}_2^+$ can be produced by the following primary charge separation mechanisms (we note, however, that these assignments do not exclude the existence of concerted or deferred charge separation pathways):

9 $$\begin{aligned} \text {C}_4\text {H}_4\text {Se}^{2+}&\rightarrow \text {C}_4\text {H}_4^+ +\, ^{80}\text {Se}^{+}/^{78}\text {SeH}_2^{+}\rightarrow \text {C}_2\text {H}_2^+ + \text {C}_2\text {H}_2/\text {C}_2 +\, ^{80}\text {Se}^{+}/^{78}\text {SeH}_2^{+}, \end{aligned}$$

10 $$\begin{aligned} \text {C}_4\text {H}_4\text {Se}^{2+}&\rightarrow \text {C}_4\text {H}_4^+ +\, ^{80}\text {Se}^{+}/^{78}\text {SeH}_2^{+}\rightarrow \text {C}_3\text {H}^+ + \text {C}\text {H}_3/\text {CH} +\, ^{80}\text {Se}^{+}/^{78}\text {SeH}_2^{+}, \end{aligned}$$

11 $$\begin{aligned} \text {C}_4\text {H}_4\text {Se}^{2+}&\rightarrow \text {C}_2\text {H}_2^+ + \text {C}_2\text {H}_2\text {Se}^{+}\rightarrow \text {C}_2\text {H}_2^+ + \text {C}_2\text {H}_2/\text {C}_2 +\, ^{80}\text {Se}^{+}/^{78}\text {SeH}_2^{+}, \end{aligned}$$

where reaction (9) corresponds to Figure 4(c), reaction (10) to (d), and reaction (11) to (e). For reactions (9) and (11), it is likely that acetylene loss is the more common occurrence due to its inherent stability, and similar outcomes have frequently been documented following the XUV ionization of aromatic hydrocarbons^16,41,42^. The non-observation of a channel producing a primary $\hbox {C}_3 \hbox {H}^+$ (e.g., Figure 4(f) exhibits no clear feature and relatively high levels of noise) again suggests that, as in Table 2, breaking two C-Se bonds is favored over breaking one C-Se and one C-C bond.

The above assignments were supported using two-step classical trajectory calculations that considered the product ions as point charges^40,43^: in the first step, the primary charge separation processes were initiated from the neutral ground-state conformation of the parent molecule, with charges placed on the center of mass of each fragment; the secondary dissociation reactions were then assumed to occur long enough after the primary process so that the two steps could be considered as isolated events. Momentum parameters describing the primary and secondary fragmentation steps were directly obtained from the covariance maps in Figure 4. For example, the average momentum of the primary $\hbox {SeH}_{y,\textrm{I}}^+$ from reaction (9) was 127$\,\pm \,$21 a.u. and that of the secondary $\hbox {C}_2 \hbox {H}_{2,\textrm{II}}^+$ was 20$\,\pm \,$10 a.u.

**Fig. 5:**
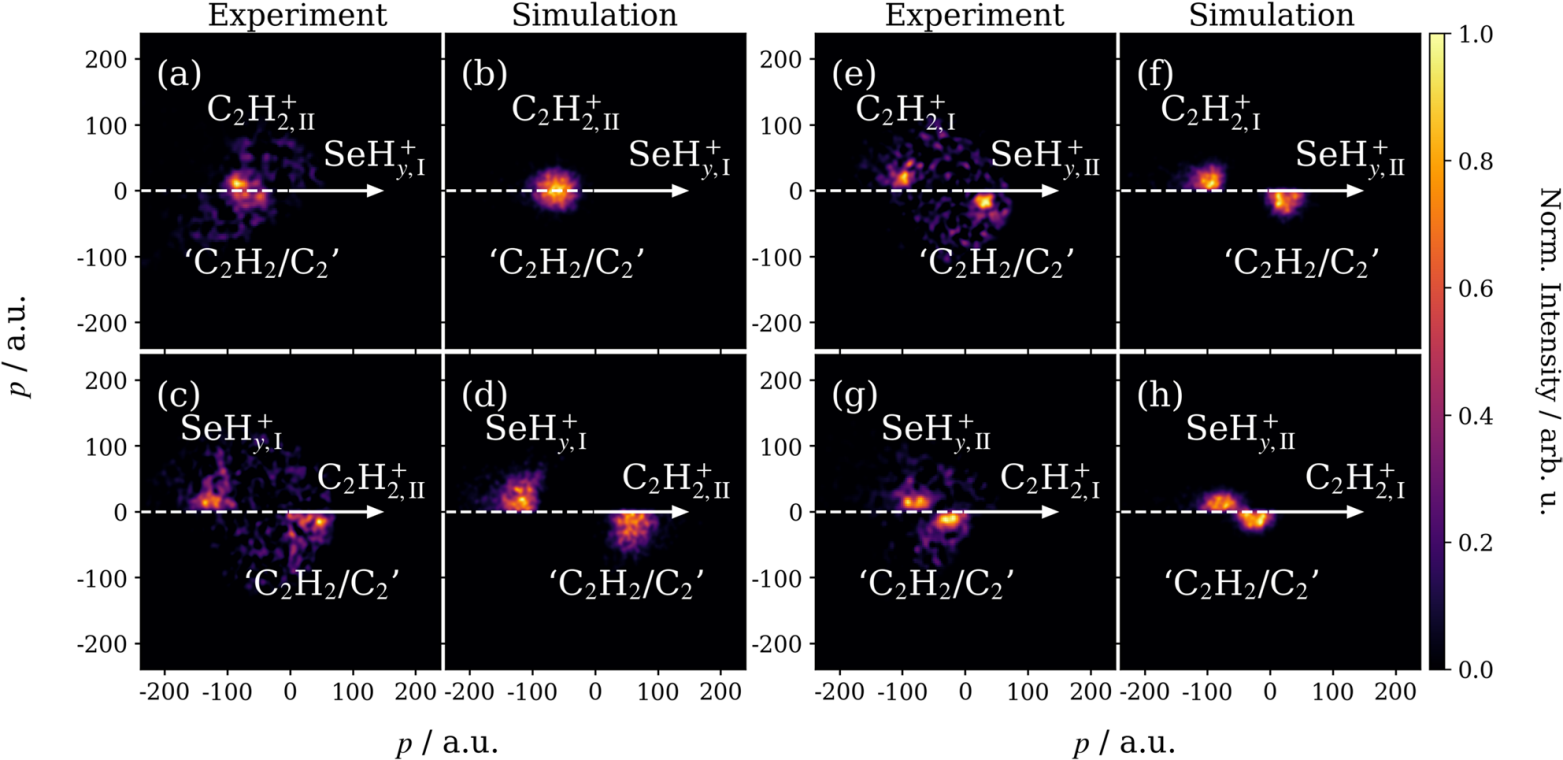
Experimental and simulated Newton-frame covariance maps of correlated $\hbox {C}_2 \hbox {H}_2^+$ and $\hbox {SeH}_{y}^+$ ($y=$ 0 or 2) momenta. The recoil distributions of secondary $\hbox {C}_2 \hbox {H}_{2,\textrm{II}}^+$ relative to primary $\hbox {SeH}_{y,\textrm{I}}^+$ are given in (**a**) and (**b**), and vice versa in (**c**) and (**d**). The recoil distributions of primary $\hbox {C}_2 \hbox {H}_{2,\textrm{I}}^+$ relative to secondary $\hbox {SeH}_{y,\textrm{II}}^+$ are given in (**e**) and (**f**), and vice versa in (**g**) and (**h**). Each Newton-frame covariance map is normalized to its own maximum, and a Gaussian blur has been added to mitigate the low signal-to-noise ratios of these channels.

Figure 5 compares the simulated outcomes of reactions (9) and (11) with the experimental results presented in Figure 4. In the former case, (a) and (c) illustrate the measured momentum distributions of $\hbox {C}_2 \hbox {H}_{2,\textrm{II}}^+$ with respect to $\hbox {SeH}_{y,\textrm{I}}^+$ and vice versa. The corresponding simulations in (b) and (d) predict virtually identical outcomes. For reaction (11), the relative momentum distributions of $\hbox {C}_2 \hbox {H}_{2,\textrm{I}}^+$ and $\hbox {SeH}_{y,\textrm{II}}^+$ in (e) and (g) are also well-characterized by the simulations. For both reactions, the simulations closely recreate the experimental recoil distributions and support their assignments as sequential mechanisms that proceed through initial charge separation steps.

### Hetero-atom substitution effects

A comparison of the selenophene fragmentation channels discussed above with those exhibited by other molecules of the form $\hbox {C}_4 \hbox {H}_4 \hbox {X}$ (X = heteroatom), such as thiophene and furan, indicates that the nature of the selenium atom significantly influences the probability distribution of reaction outcomes. For example, Kukk *et al.* carried out a similar experiment on thiophene ($\hbox {C}_4 \hbox {H}_4 \hbox {S}$) using 191 eV light, which predominantly ionized the sulfur 2p orbital to produce molecular dications through an Auger-Meitner process^17,18^. The heteroatom site-selectivity of their experiment had a similar order of magnitude to the present work on selenophene, and also greater selectivity for the +2 charge state. Table 3 demonstrates that ionization of thiophene at the sulfur 2p orbital is expected to occur 84% of the time at 191 eV, whereas the analogous value for the selenium 3 d orbital is 78% at 120 eV^44^. The ratio of di- to trication production for atomic sulfur is approximately 8:1, using the photoionization cross-sections just above the argon 2p edge as a reference, and is about 6:1 above the sulfur 2p edge in carbonyl sulfide and carbon disulfide^37,45^. By contrast, the same ratio is 2.5:1 for selenium at 120 eV. The thiophene dications were observed to overwhelmingly fragment through two-body mechanisms to create $\hbox {C}_n \hbox {H}_x^{+}$/$\hbox {C}_{(4-n)} \hbox {H}_y \hbox {X}^{+}$ ion pairs, of which the most probable outcome by far was the formation of $\hbox {C}_3 \hbox {H}_x^{+}$ and $\hbox {CH}_y \hbox {X}^{+}$, followed by $\hbox {C}_2 \hbox {H}_x^+$ and $\hbox {C}_2 \hbox {H}_y \hbox {X}^+$. A many-body fragmentation channel producing $\hbox {X}^{+}$, $\hbox {C}_2 \hbox {H}_2^+$ and $\hbox {C}_2 \hbox {H}_2$ was also observed, similar to that described in Figure 4, but was much less intense than the two-body outcomes. By contrast, the most prominent channels observed for selenophene produce $\hbox {C}_n \hbox {H}_x^{+}$ ($n<4$) and $\hbox {XH}_y^+$ with a second (likely neutral) carbon-containing product. The two-body $\hbox {C}_n \hbox {H}_x^{+}$/$\hbox {C}_{(4-n)} \hbox {H}_y \hbox {X}^{+}$ mechanisms (Table 2) that are analogous to the thiophene channels occur less frequently, and, of these, the $\hbox {C}_3 \hbox {H}_x^{+}$ and $\hbox {C}_2 \hbox {H}_x^+$ channels only yield 32% and 14% of their total intensity, while $\hbox {C}_4 \hbox {H}_x^{+}$ channels contribute 51%. This implies that the dissociation of one C-X and one C-C bond is more favorable in thiophene than selenophene, whereas the latter tends to break two C-X bonds.

Turning to furan, Pešić *et al.* investigated the fragmentation dynamics of the molecular dication following core ionization of the carbon and oxygen 1s orbitals at 535.2 eV^19^. The similar ionization cross sections of these orbitals rule out a purely site-selective ionization experiment (see Table 3), but the results are still generally in line with the preceding discussion. In this case, various many-body fragmentation pathways were observed, including intense channels producing the $\hbox {C}_3 \hbox {H}_x^{+}$ and $\hbox {CH}_y \hbox {X}^{+}$ fragments that were the dominant outcome seen for the breakup of the thiophene dication. The fragmentation of furan dications generated by electron impact valence ionization has also been investigated by Heathcote *et al.*^20^. Similar to thiophene, the most significant outcomes in both furan experiments were again $\hbox {C}_3 \hbox {H}_x^+$/$\hbox {CH}_y \hbox {X}^{+}$ ion pairs, followed by $\hbox {C}_2 \hbox {H}_x^+$/$\hbox {C}_2 \hbox {H}_y \hbox {X}^+$ products. In contrast to selenophene, no correlations were observed between $\hbox {C}_4 \hbox {H}_x^+$ and $\hbox {XH}_y^+$. As such, the behavior of furan with respect to selenophene is very similar to that of thiophene: as the size of the heteroatom is reduced, the probability of breaking both C-X bonds decreases, and the probability of breaking one C-X and one C-C bond increases.

A qualitative explanation for the differences observed between selenophene and the other heterocyclic systems is that carbon-selenium bonds are about 60–80% as strong as carbon-sulfur and carbon-oxygen bonds^38^. For example, the dissociation of selenium from selenophene ($\hbox {C}_4 \hbox {H}_4$ + Se) requires approximately 390 kJ $\hbox {mol}^{-1}$, but this rises to 480 kJ $\hbox {mol}^{-1}$ for the analogous thiophene reaction^39,46^. As such, when solely considering these energies, one would expect a larger yield of $\hbox {C}_4 \hbox {H}_x^+$ and $\hbox {XH}_y^+$ products from the selenophene dication than from thiophene or furan. This explanation is not complete, however, as the latter two dications primarily cleave one C-X bond and one C-C bond upon dissociation, even though breaking two of the former would require less energy. It is conceivable that, relative to selenophene, the stronger valence orbital overlap between the heteroatom and the carbon framework of these molecules helps facilitate charge redistribution following the Auger-Meitner process, potentially making it easier to break a C-C bond. It should be emphasized that, although this interpretation is consistent with the observed results, it does not take into account the distinct potential energy surface landscapes of these molecules or the possibility of double valence ionization occurring at alternative sites to the heteroatom. The relative probabilities of different reaction mechanisms and the dependence on the heteroatom are influenced by a complex interplay between the electronic structures, primary and secondary ionization processes, charge redistribution mechanisms, and excited state relaxation dynamics involved. These are beyond the scope of the present work, but we note that some of these factors may potentially be distinguishable by measuring the Auger-Meitner electrons associated with the above processes.

**Table 3:** The heteroatom site-selectivity of selenophene, thiophene and furan at various photon energies, as determined by the Se 3d, S 2p and O 1s orbital cross sections ($\sigma _{\text {X}\,{nl}}$, where X is the heteroatom, *n* is the energy level, and *l* is the orbital type), and by the total heteroatom cross sections ($\sigma _{\text {X}}$, including all accessible atomic orbitals of the heteroatom at the given photon energy)^44^. The preference for site-selective ionization is expressed as a fraction of the value expected for the whole molecule ($\Sigma \sigma _{\text {C}_4\text {H}_4\text {X}}$, where the sum represents the accessible atomic orbitals of the constituent atoms at the given photon energy).

Molecule	$\hbox {C}_4 \hbox {H}_4$X	X *nl*	Photon energy/eV	$\sigma _{\text {X}\,{nl}}$/$\Sigma \sigma _{\text {C}_4\text {H}_4\text {X}}$	$\Sigma \sigma _\text {X}$/$\Sigma \sigma _{\text {C}_4\text {H}_4\text {X}}$
Selenophene	$\hbox {C}_4 \hbox {H}_4$Se	Se 3d	120	0.78	0.81
Thiophene^17^	$\hbox {C}_4 \hbox {H}_4$S	S 2p	191	0.84	0.88
Furan^19^	$\hbox {C}_4 \hbox {H}_4$O	O 1s	535.2	0.36	0.38

## Discussion

The inner-shell ionization of selenophene at 120 eV gives rise to two major fragmentation pathways: those producing $\hbox {C}_n \hbox {H}_x^{+}$, $\hbox {Se}^+$/$\hbox {SeH}_y^+$, and a second carbon-containing fragment; and those producing $\hbox {C}_n \hbox {H}_x^{+}$ and $\hbox {C}_{(4-n)} \hbox {H}_y \hbox {Se}^{+}$. The latter grouping includes more than 50 fragmentation channels, at least 25% of which could be clearly attributed to various two-body separations of the parent dication following single-photon absorption. The initial selenophene charge states of the remaining many-body processes could not be definitively assigned, due to the prevalence of undetectable $\hbox {H}^{0/+}$ or neutral carbon species, but were in general consistent with the double or triple ionization of selenophene. Since single-photon absorption by isolated selenium atoms at 120 eV results in +2 and +3 charge states in a roughly 2.5:1 ratio, these results suggest that a broad majority of the observed reaction pathways resulted from single-photon ionization.

The above reaction channels produced large numbers of similar fragments that differed only by their selenium isotope or number of hydrogen atoms. These were disentangled using covariance analysis, which used the relative momenta of particular ion pairs to assign each channel. This further enabled the relative yields of the $\hbox {C}_n \hbox {H}_x^{+}$/$\hbox {C}_{(4-n)} \hbox {H}_y \hbox {Se}^{+}$ channels to be estimated to a reasonable degree of accuracy. The results indicate that selenophene fragmentation at 120 eV tends to break both C-Se bonds. By contrast, the analogous heterocycles thiophene and furan dissociate by cleaving one C-C bond and one C-X bond^17–20^. This can in part be attributed to the relative bond strengths involved, but it may also be indicative of how easily charge redistribution can occur following the Auger-Meitner process. In this case, the relatively weaker valence orbital overlap between selenium and the carbon framework may mean more electron density is localized on the selenium atom, potentially weakening the C-Se bonds^7,47^.

Overall, the present study demonstrates that photoion-photoion covariance analysis is a powerful tool for distinguishing and comparing the large numbers of fragmentation pathways that can be initiated by inner-shell ionization and valence electron Auger-Meitner decay. Interpreting such outcomes is a necessary step in applying XUV and X-ray spectroscopy in combination with CEI-MS to investigate the structures and dynamics of complex systems, and the results presented here show that even fragmentation channels producing ions with the same *m*/*z* but different chemical compositions can be differentiated. Combining CEI-MS with coincident Auger-Meitner electron measurements may therefore offer a viable route to more detailed investigations of molecular fragmentation dynamics, particularly in cases where photoelectron kinetic energies can be assigned to specific primary ionization processes.

## Methods

### Experiment

Selenophene fragment ion momenta were recorded at the soft X-ray beamline (BL1) of SACLA using the velocity-map imaging spectrometer developed by Ueda and coworkers^27–29,43,48^. A pulsed molecular beam of neat selenophene was expanded into the VMI chamber and crossed at a $45^{\circ }$ angle by a 60 Hz beam of 120 eV photons (bandwidth = 2%) produced by SACLA. The laser pulse duration was approximately 30 fs^49^. Pulse energies were measured shot-by-shot using a gas intensity monitor and determined to have a Gaussian mean and standard deviation of 11.3 ± 1.1 $\mu$J. The laser beam was attenuated to 1.9 ± 0.2 $\mu$J in the VMI chamber due to the combined transmission efficiencies of the beamline (90%) and a 0.5 $\mu$m zirconium filter (18.6%) placed in the beam path. A histogram of attenuated pulse energies is given in Figure S20 of the Supplementary Information. The beam was focused using a Kirkpatrick–Baez mirror to a spot size of 10 $\mu$m (1/$\textrm{e}^2$), yielding a Gaussian intensity of roughly 1.5 $\times$ $10^{14}$ W $\hbox {cm}^{-2}$ in the VMI spectrometer interaction region.

The absorption cross sections of several selenium, carbon, and hydrogen orbitals at 120 eV are given in Table 4^44^. The absorption cross section ratio of the selenium 3d orbital with respect to all other selenophene orbitals ionizable at this energy is approximately 3.5:1, indicating a strong preference for site-selective absorption. Based on the behavior of isolated krypton atoms, which are electronically similar to selenium, single-photon absorption by the Se 3d orbital at 120 eV should predominantly result in double or triple ionization through Auger-Meitner decay in a 2.5:1 ratio^6,37^. This is consistent with the results presented here, which suggest that selenophene is primarily ionized to its +2 and +3 charge states before fragmenting.

The selenophene parent and fragment ions generated following inner-shell ionization were subsequently accelerated by VMI potential fields, separated by their times-of-flight, and electrostatically mapped onto a dual microchannel plate (MCP) array coupled to a hexanode delay line detector^48^. Roughly 17 ions were detected per laser shot, however these were limited to mass-to-charge ratios of 5 or more due to the presence of scattered light on the detector. This meant that individual protons could not reliably be detected in these experiments. Three-dimensional momenta were reconstructed for each ion hit by using the detector *x* and *y* coordinates to calculate their corresponding momentum components perpendicular to the time-of-flight axis ($\vec {p}_x$, $\vec {p}_y$), and the ion times-of-flight to calculate the remaining component ($\vec {p}_z$). The momentum resolution was found to be 8 a.u. (atomic units, defined as $\hbar$/$\hbox {a}_0$ where 1 a.u. = 1.993 $\times$ $10^{-24}$ kg m $\hbox {s}^{-1}$)^40^.

**Table 4:** Calculated absorption cross sections ($\sigma$) at 120 eV for orbitals belonging to the constituent atoms of selenophene^44^.

Orbital	$\sigma$ (Mb)	Orbital	$\sigma$ (Mb)	Orbital	$\sigma$ (Mb)
Se 3d	6.13	C 2p	0.10	H 1s	0.01
Se 4p	0.11	C 2s	0.27		
Se 4s	0.12				

### Covariance analysis

Newton-frame covariance mapping was used to gauge the relative populations of the fragmentation pathways discussed in the Results section^43,50^. The three-dimensional momentum information associated with specified ion pairs was first used to calculate their covariant momentum distributions, as shown in Figure 2(a). The intensities of notable features in the resulting covariance images, integrated over angle and momentum, were then taken as a measure of how often they occurred (assuming similar detection efficiencies for each ion)^30^. These values and their uncertainties were evaluated using an adapted bootstrapping method^16^. This involved determining the integrated covariance intensities of a given ion pair individually for 100 subsets of 100,000 laser shots, randomly selected from the 1,910,241 acquired, and fitting the resulting values to determine their Gaussian mean and standard deviation.

Due to the congestion of the mass spectrum in Figure 1, which is primarily caused by the close grouping of fragments that differ by the selenium isotope involved or the number of hydrogen atoms, the momentum information of several ion species used in the above analysis was limited to specific ranges to avoid introducing false correlations with ions from neighboring time-of-flight peaks. These considerations are detailed in Section 6 of the Supplementary Information. Additionally, the contingent covariance method was applied to further minimize false correlations caused by shot-to-shot fluctuations in the free electron laser intensity^31,43,51,52^. This involved splitting the pulse energy data into ten equal bins (as shown in Figure S20 of the Supplementary Information), calculating the Newton-frame covariance maps associated with the data in each bin, and then averaging the results to produce the images that were subsequently used to determine the integrated intensities discussed above.

## Supplementary Information

Supplementary Information.

## Data Availability

The dataset presented in this report is publicly hosted by the UK STFC at https://doi.org/10.5286/edata/961.
