# Supplementary material for: The inner-shell ionization and fragmentation of selenophene at 120 eV
Source: Sci Rep. 2026 Feb 17;16:9442. doi: 10.1038/s41598-026-39246-4 (PMC13004873; doi:10.1038/s41598-026-39246-4)
Supplement: Supplementary file 1 — Supplementary Information. [file 41598_2026_39246_MOESM1_ESM.pdf]

Supplementary information for: The inner-shell ionization and fragmentation of selenophene at 120 eV

Tiffany Walmsley,[1,2,*] Felix Allum,[3,4,5] James R. Harries,[6] Yoshiaki Kumagai,[7,8] Joseph W. McManus,[2] Kiyonobu Nagaya,[9] Mathew Britton,[3,4] Mark Brouard,[2] Philip Bucksbaum,[4] Mizuho Fushitani,[10] Ian Gabalski,[4,11] Tatsuo Gejo,[12] Paul Hockett,[13] Andrew J. Howard,[4,11] Hiroshi Iwayama,[14] Edwin Kukk,[15] Chow-shing Lam,[2] Russell S. Minns,[16] Akinobu Niozu,[17] Sekito Nishimuro,[18] Johannes Niskanen,[15] Shigeki Owada,[19,20] Weronika O. Razmus,[16] Daniel Rolles,[21] James D. Somper,[2] Kiyoshi Ueda,[22] James Unwin,[2] Shin-ichi Wada,[23] Joanne L. Woodhouse,[16] Ruaridh Forbes,[3,24] Michael Burt,[2,25,*] and Emily M. Warne[2]

[1] *Central Laser Facility, STFC Rutherford Appleton Laboratory, Didcot OX11 0QX, United Kingdom*
[2] *Chemistry Research Laboratory, Department of Chemistry, University of Oxford, Oxford OX1 3TA, United Kingdom*
[3] *Linac Coherent Light Source, SLAC National Accelerator Laboratory, 2575 Sand Hill Road, Menlo Park, CA 94025, USA*
[4] *PULSE Institute, SLAC National Accelerator Laboratory, 2575 Sand Hill Road, Menlo Park, CA 94025, USA*
[5] *Deutsches Elektronen-Synchrotron, DESY, Notkestr. 85, 22607, Hamburg, Germany*
[6] *National Institutes for Quantum Science and Technology (QST), SPring-8, 1-1-1 Kouto, Sayo, Hyogo 679-5148, Japan*
[7] *Department of Applied Physics, Tokyo University of Agriculture and Technology, Tokyo, Japan*
[8] *Department of Physics, Nara Women's University, Nara, 630-8506, Japan*
[9] *Department of Physics, Kyoto University, Kyoto 606-8502, Japan*
[10] *Department of Chemistry, Graduate School of Science, Nagoya University, Nagoya, Aichi 464-8602, Japan*
[11] *Department of Applied Physics, Stanford University, Stanford, CA 94305-4090, USA*
[12] *Graduate School of Material Science, University of Hyogo, Kouto 3-2-1, Kamigori-cho, Ako-gun, Hyogo 678-1297, Japan*
[13] *National Research Council of Canada, 100 Sussex Dr. Ottawa, ON K1A 0R6, Canada*
[14] *Institute for Molecular Science, Okazaki 444-8585, Japan*
[15] *Department of Physics and Astronomy, University of Turku, FI-20014 Turku, Finland*
[16] *School of Chemistry, University of Southampton, Highfield, Southampton SO17 1BJ, United Kingdom*
[17] *Graduate School of Humanities and Social Sciences, Hiroshima University, Higashi-Hiroshima 739-8524, Japan*
[18] *Department of Materials Science and Engineering, School of Materials and Chemical Technology, Institute of Science Tokyo, 2-12-1 W4-10 Ookayama, Meguro-ku, Tokyo 152-8550, Japan*
[19] *Japan Synchrotron Radiation Research Institute, Kouto 1-1-1 Sayo, Hyogo, Japan*
[20] *RIKEN SPring-8 Center, Kouto 1-1-1 Sayo, Hyogo, Japan*
[21] *J. R. Macdonald Laboratory, Department of Physics, Kansas State University, Manhattan, Kansas 66506, USA*
[22] *Department of Chemistry, Tohoku University, Sendai 980-8578, Japan*
[23] *Graduate School of Advanced Science and Engineering, Hiroshima University, Higashi-Hiroshima 739-8526, Japan*
[24] *Department of Chemistry, University of California, Davis, One Shields Avenue, Davis, CA 95616, USA*
[25] *Department of Chemistry, Trent University, Peterborough, ON K9L 0G2, Canada*

* *Contact Emails: tiffany.walmsley@stfc.ac.uk and michaelburt@trentu.ca*

October 2025

1 Isotope percentages from covariances

The relative yields of all channels producing $C_nH_x^+$ and $C_{(4-n)}H_ySe^+$, with $n = 1 - 4$ and $x + y \leq 4$, are provided in the following figures and tables:

- $C_4H_x{}^+$ + $SeH_y{}^+$: Figures S1-S5; Tables S1-S5
- $C_3H_x{}^+$ + CH_ySe^+: Figures S6-S10; Tables S6-S10
- $C_2H_x{}^+$ + $C_2H_ySe^+$: Figures S11-S14; Tables S11-S14
- $CH_x{}^+$ + $C_3H_ySe^+$: Figures S15-S18; Tables S15-S18.

These were determined using the methodology described in the Results section of the main text. Briefly, the integrated intensities of any features observed in the $C_nH_x^+/C_{(4-n)}H_ySe^+$ Newton-frame covariance maps could in general be assigned to reactions (5) and (6) in the paper. In instances where a covariance feature could be assigned to multiple channels, the intensities of these pathways were apportioned using the relative abundances of the selenium isotopes that could be involved, assuming there was no likelihood of isotope-specific chemistry. For example, the $C_4^+/^{78}SeH_y^+$ covariance in Table S1 has three possible assignments:

$$C_4H_4Se^{2/3+} \rightarrow C_4^+ + {}^{78}Se^+ + 4H^{0/+}, \tag{1}$$

$$C_4H_4Se^{2/3+} \rightarrow C_4^+ + {}^{77}SeH^+ + 3H^{0/+}, \tag{2}$$

$$C_4H_4Se^{2/3+} \rightarrow C_4^+ + {}^{76}SeH_2^+ + 2H^{0/+}. \tag{3}$$

Reactions (1) to (3) were therefore assumed to occur 23%, 19% and 59% of the time, respectively, based on the expected amounts of ^{76}Se, ^{77}Se and ^{78}Se. As outlined in the main text, this presumes that all fragmentation channels associated with a covariant $C_nH_x^+/C_{(4-n)}H_ySe^+$ pair occur with equal probability. Although this almost certainly over- or underestimates contributions from individual channels, a comparison of the total isotope yields for each $C_nH_x^+/C_{(4-n)}H_ySe^+$ pair demonstrates that, in general, they match the natural isotopic distribution of selenium. This is a strong indicator that this simple assumption is reasonable.

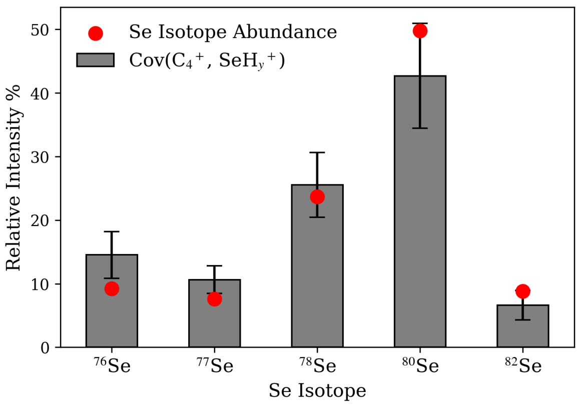

Figure S1: The summed relative intensities of fragmentation channels producing C_4^+ and all SeH_y^+ co-fragments incorporating the different selenium isotopes, also given in Table S1. These covariances made up $4.7 \pm 0.5\%$ of all covariances observed for the $C_nH_x^+ + C_{(4-n)}H_ySe^+$ (where $x + y \leq 4$) fragmentation channels. The error bars correspond to uncertainty values obtained from an adapted bootstrapping method outlined in the Methods section. Red dots indicate the natural selenium isotope abundances. The expected Se isotope abundances are also given by red dots.

SeH_y^+ m/z	Intensity (arb. u.)	^{76}Se (9.2%)	^{77}Se (7.6%)	^{78}Se (23.7%)	^{80}Se (49.8%)	^{82}Se (8.8%)
76	0.9 ± 0.4	0.9 ± 0.4				
77	1.3 ± 0.5	0.7 ± 0.3	0.6 ± 0.2			
78	2.3 ± 0.9	0.5 ± 0.2	0.4 ± 0.2	1.3 ± 0.5		
79	2.2 ± 0.6		0.5 ± 0.2	1.7 ± 0.5		
80	2.3 ± 0.7			0.7 ± 0.2	1.6 ± 0.5	
81	3.6 ± 1.1				3.6 ± 1.1	
82	1.3 ± 0.4				1.1 ± 0.4	0.2 ± 0.1
83	0.8 ± 0.3					0.8 ± 0.3
	Intensity (%)	14.5 ± 3.7	10.6 ± 2.2	25.6 ± 5.1	42.7 ± 8.3	6.6 ± 2.3

Table S1: The integrated intensities of Newton frame covariance maps between C_4^+ and SeH_y^+ fragments ($m/z = 76 - 83\,u$). Note that no covariance was observed between C_4^+ and SeH_y^+ where $m/z = 84\,u$.

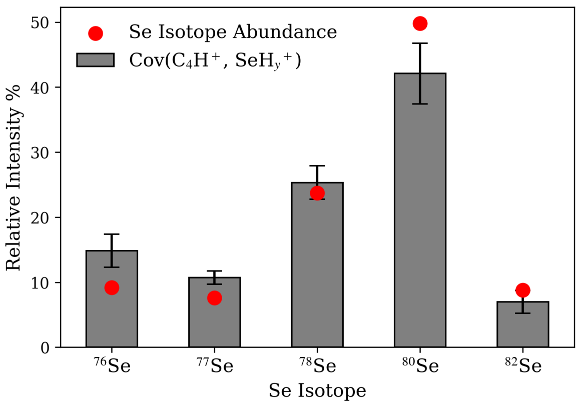

Figure S2: The summed relative intensities of fragmentation channels producing C_4H^+ and all $SeH_y{}^+$ co-fragments incorporating the different selenium isotopes, also given in Table S2. These covariances made up $12.3 \pm 0.7\%$ of all covariances observed for the $C_nH_x{}^+ + C_{(4-n)}H_ySe^+$ (where $x + y \leq 4$) fragmentation channels. The error bars correspond to uncertainty values obtained from an adapted bootstrapping method outlined in the Methods section. Red dots indicate the natural selenium isotope abundances. The expected Se isotope abundances are also given by red dots.

$SeH_y{}^+$ m/z	Intensity (arb. u.)	^{76}Se (9.2%)	^{77}Se (7.6%)	^{78}Se (23.7%)	^{80}Se (49.8%)	^{82}Se (8.8%)
76	2.2 ± 0.9	2.2 ± 0.9				
77	4.1 ± 0.6	2.2 ± 0.3	1.9 ± 0.3			
78	5.4 ± 0.9	1.2 ± 0.2	1.0 ± 0.2	3.1 ± 0.5		
79	5.0 ± 1.0		1.2 ± 0.2	3.8 ± 0.7		
80	8.4 ± 1.1			2.7 ± 0.4	5.7 ± 0.8	
81	7.0 ± 1.5				7.0 ± 1.5	
82	4.0 ± 0.6				3.4 ± 0.5	0.6 ± 0.1
83	1.6 ± 0.6					1.6 ± 0.6
84	0.4 ± 0.3					0.4 ± 0.3
	Intensity (%)	14.9 ± 2.5	10.7 ± 1.0	25.3 ± 2.6	42.1 ± 4.7	7.0 ± 1.8

Table S2: The integrated intensities of Newton frame covariance maps between C_4H^+ and $SeH_y{}^+$ fragments ($m/z = 76 - 84\,u$).

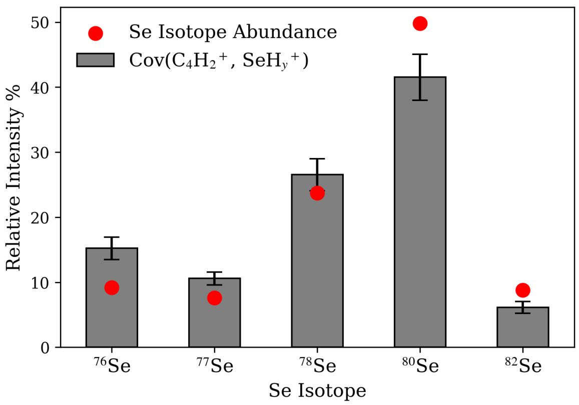

Figure S3: The summed relative intensities of fragmentation channels producing $C_4H_2^+$ and all SeH_y^+ co-fragments incorporating the different selenium isotopes, also given in Table S3. These covariances made up $20.6 \pm 1.9\%$ of all covariances observed for the $C_nH_x^+ + C_{(4-n)}H_ySe^+$ (where $x + y \leq 4$) fragmentation channels. The error bars correspond to uncertainty values obtained from an adapted bootstrapping method outlined in the Methods section. Red dots indicate the natural selenium isotope abundances. The expected Se isotope abundances are also given by red dots.

SeH_y^+ m/z	Intensity (arb. u.)	^{76}Se (9.2%)	^{77}Se (7.6%)	^{78}Se (23.7%)	^{80}Se (49.8%)	^{82}Se (8.8%)
76	4.0 ± 0.9	4.0 ± 0.9				
77	6.1 ± 1.0	3.3 ± 0.5	2.8 ± 0.4			
78	10.2 ± 1.6	2.3 ± 0.4	1.9 ± 0.3	6.0 ± 0.9		
79	8.3 ± 1.3		2.0 ± 0.3	6.3 ± 1.0		
80	14.0 ± 2.3			4.5 ± 0.7	9.5 ± 1.5	
81	9.8 ± 1.2				9.8 ± 1.2	
82	8.2 ± 1.2				6.9 ± 1.1	1.2 ± 0.2
83	2.0 ± 0.4					2.0 ± 0.4
84	0.6 ± 0.4					0.6 ± 0.4
	Intensity (%)	15.2 ± 1.7	10.7 ± 1.0	26.5 ± 2.5	41.5 ± 3.5	6.1 ± 0.9

Table S3: The integrated intensities of Newton frame covariance maps between $C_4H_2^+$ and SeH_y^+ fragments ($m/z = 76 - 84\,u$).

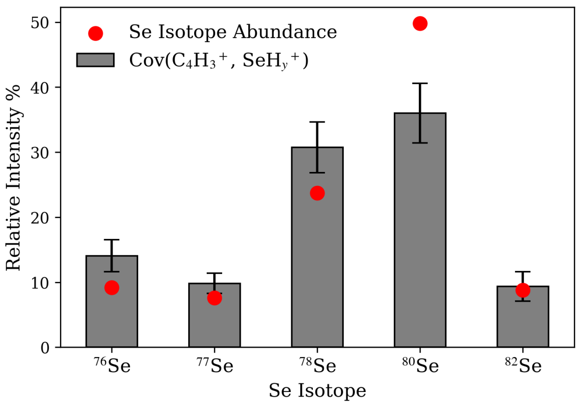

Figure S4: The summed relative intensities of fragmentation channels producing $C_4H_3{}^+$ and all $SeH_y{}^+$ co-fragments incorporating the different selenium isotopes, also given in Table S4. These covariances made up $12.0 \pm 0.8\%$ of all covariances observed for the $C_nH_x{}^+ + C_{(4-n)}H_ySe^+$ (where $x + y \leq 4$) fragmentation channels. The error bars correspond to uncertainty values obtained from an adapted bootstrapping method outlined in the Methods section. Red dots indicate the natural selenium isotope abundances. The expected Se isotope abundances are also given by red dots.

$SeH_y{}^+$ m/z	Intensity (arb. u.)	^{76}Se (9.2%)	^{77}Se (7.6%)	^{78}Se (23.7%)	^{80}Se (49.8%)	^{82}Se (8.8%)
76	2.7 ± 0.6	2.7 ± 0.6				
77	4.3 ± 1.1	2.4 ± 0.6	1.9 ± 0.5			
78	6.5 ± 1.1		1.6 ± 0.3	4.9 ± 0.9		
79	6.1 ± 1.1			6.1 ± 1.1		
80	6.1 ± 1.1				6.1 ± 1.1	
81	6.8 ± 1.2				6.8 ± 1.2	
82	2.2 ± 0.7					2.2 ± 0.7
83	1.1 ± 0.4					1.1 ± 0.4
	Intensity (%)	14.1 ± 2.5	9.8 ± 1.6	30.8 ± 3.9	36.0 ± 4.6	9.3 ± 2.3

Table S4: The integrated intensities of Newton frame covariance maps between $C_4H_3{}^+$ and $SeH_y{}^+$ fragments ($m/z = 76 - 83\,u$).

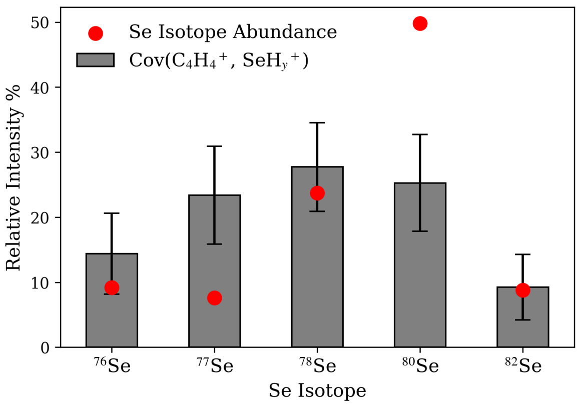

Figure S5: The summed relative intensities of fragmentation channels producing $C_4H_4^+$ and all SeH_y^+ co-fragments incorporating the different selenium isotopes, also given in Table S5. These covariances made up $2.7 \pm 0.4\%$ of all covariances observed for the $C_nH_x^+ + C_{(4-n)}H_ySe^+$ (where $x + y \leq 4$) fragmentation channels. The error bars correspond to uncertainty values obtained from an adapted bootstrapping method outlined in the Methods section. Red dots indicate the natural selenium isotope abundances. The expected Se isotope abundances are also given by red dots.

SeH_y^+ m/z	Intensity (arb. u.)	^{76}Se (9.2%)	^{77}Se (7.6%)	^{78}Se (23.7%)	^{80}Se (49.8%)	^{82}Se (8.8%)
76	1.1 ± 0.5	1.1 ± 0.5				
77	1.8 ± 0.6		1.8 ± 0.6			
78	2.2 ± 0.5			2.2 ± 0.5		
79	-					
80	2.0 ± 0.6				2.0 ± 0.6	
81	-					
82	0.7 ± 0.4					0.7 ± 0.4
	Intensity (%)	14.4 ± 6.2	23.4 ± 7.5	27.7 ± 6.8	25.3 ± 7.4	9.2 ± 5.0

Table S5: The integrated intensities of Newton frame covariance maps between $C_4H_4^+$ and SeH_y^+ fragments ($m/z = 76 - 82\,u$).

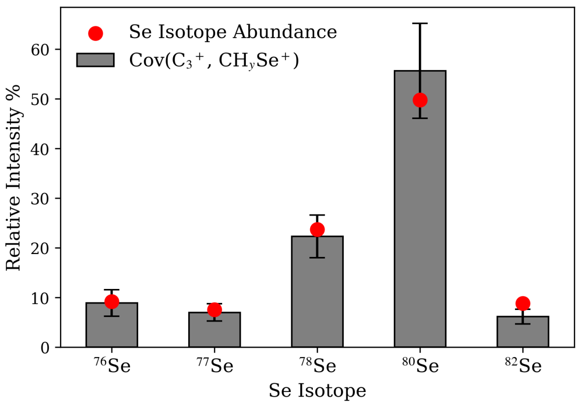

Figure S6: The summed relative intensities of fragmentation channels producing C_3^+ and all CH_ySe^+ co-fragments incorporating the different selenium isotopes, also given in Table S6. These covariances made up $3.5 \pm 0.4\%$ of all covariances observed for the $C_nH_x{}^+ + C_{(4-n)}H_ySe^+$ (where $x + y \leq 4$) fragmentation channels. The error bars correspond to uncertainty values obtained from an adapted bootstrapping method outlined in the Methods section. Red dots indicate the natural selenium isotope abundances. The expected Se isotope abundances are also given by red dots.

CH_ySe^+ m/z	Intensity (arb. u.)	^{76}Se (9.2%)	^{77}Se (7.6%)	^{78}Se (23.7%)	^{80}Se (49.8%)	^{82}Se (8.8%)
88	0.2 ± 0.2	0.2 ± 0.2				
89	0.4 ± 0.3	0.2 ± 0.2	0.2 ± 0.1			
90	0.8 ± 0.4	0.2 ± 0.1	0.2 ± 0.1	0.5 ± 0.2		
91	1.4 ± 0.5	0.3 ± 0.1	0.3 ± 0.1	0.8 ± 0.3		
92	1.5 ± 0.5		0.1 ± 0.05	0.4 ± 0.1	0.9 ± 0.3	
93	1.9 ± 0.6			0.6 ± 0.2	1.3 ± 0.4	
94	1.9 ± 0.5				1.6 ± 0.5	0.3 ± 0.1
95	2.4 ± 0.9				2.1 ± 0.8	0.4 ± 0.1
	Intensity (%)	8.9 ± 2.7	7.0 ± 1.8	22.3 ± 4.3	55.6 ± 9.6	6.2 ± 1.5

Table S6: The integrated intensities of Newton frame covariance maps between C_3^+ and CH_ySe^+ fragments ($m/z = 88 - 95\,u$). No covariance was observed for $m/z > 95\,u$.

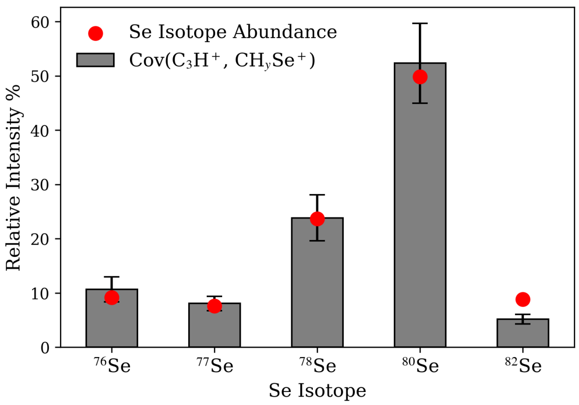

Figure S7: The summed relative intensities of fragmentation channels producing C_3H^+ and all CH_ySe^+ co-fragments incorporating the different selenium isotopes, also given in Table S7. These covariances made up $5.4 \pm 0.4\%$ of all covariances observed for the $C_nH_x{}^+ + C_{(4-n)}H_ySe^+$ (where $x + y \leq 4$) fragmentation channels. The error bars correspond to uncertainty values obtained from an adapted bootstrapping method outlined in the Methods section. Red dots indicate the natural selenium isotope abundances. The expected Se isotope abundances are also given by red dots.

CH_ySe^+ m/z	Intensity (arb. u.)	^{76}Se (9.2%)	^{77}Se (7.6%)	^{78}Se (23.7%)	^{80}Se (49.8%)	^{82}Se (8.8%)
88	0.4 ± 0.3	0.4 ± 0.3				
89	0.9 ± 0.2	0.5 ± 0.1	0.4 ± 0.1			
90	1.3 ± 0.6	0.3 ± 0.1	0.2 ± 0.1	0.8 ± 0.4		
91	2.1 ± 0.7	0.5 ± 0.2	0.4 ± 0.1	1.2 ± 0.4		
92	2.4 ± 0.6		0.2 ± 0.1	0.7 ± 0.1	1.5 ± 0.4	
93	3.2 ± 1.1			1.0 ± 0.3	2.1 ± 0.7	
94	1.8 ± 0.6				1.5 ± 0.5	0.3 ± 0.1
95	3.6 ± 0.8				3.1 ± 0.6	0.5 ± 0.1
	Intensity (%)	10.6 ± 2.3	8.1 ± 1.3	23.8 ± 4.2	52.3 ± 7.3	5.2 ± 0.9

Table S7: The integrated intensities of Newton frame covariance maps between C_3H^+ and CH_ySe^+ fragments ($m/z = 88 - 95\,u$). No covariance was observed for $m/z > 95\,u$.

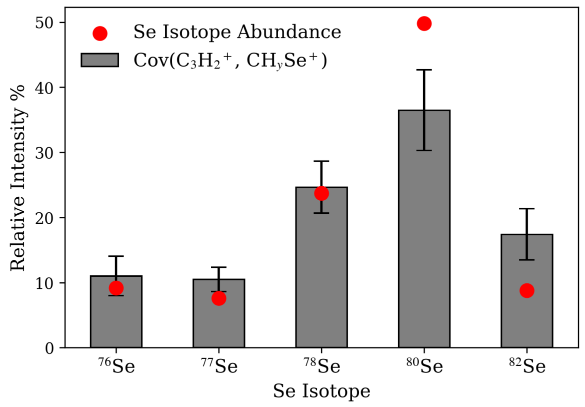

Figure S8: The summed relative intensities of fragmentation channels producing $C_3H_2^+$ and all CH_ySe^+ co-fragments incorporating the different selenium isotopes, also given in Table S8. These covariances made up $5.5 \pm 0.5\%$ of all covariances observed for the $C_nH_x{}^+ + C_{(4-n)}H_ySe^+$ (where $x + y \leq 4$) fragmentation channels. The error bars correspond to uncertainty values obtained from an adapted bootstrapping method outlined in the Methods section. Red dots indicate the natural selenium isotope abundances. The expected Se isotope abundances are also given by red dots.

CH_ySe^+ m/z	Intensity (arb. u.)	^{76}Se (9.2%)	^{77}Se (7.6%)	^{78}Se (23.7%)	^{80}Se (49.8%)	^{82}Se (8.8%)
88	0.6 ± 0.4	0.6 ± 0.4				
89	1.4 ± 0.5	0.8 ± 0.3	0.6 ± 0.2			
90	1.9 ± 0.6	0.4 ± 0.1	0.4 ± 0.1	1.1 ± 0.3		
91	3.0 ± 0.7		0.7 ± 0.2	2.3 ± 0.5		
92	2.1 ± 0.7			0.7 ± 0.2	1.4 ± 0.5	
93	3.8 ± 0.8				3.8 ± 0.8	
94	0.9 ± 0.4				0.7 ± 0.3	0.1 ± 0.1
95	2.7 ± 0.6					2.7 ± 0.6
	Intensity (%)	11.0 ± 3.0	10.5 ± 1.9	24.6 ± 4.0	36.5 ± 6.2	17.4 ± 3.9

Table S8: The integrated intensities of Newton frame covariance maps between $C_3H_2^+$ and CH_ySe^+ fragments ($m/z = 88 - 95\,u$). No covariance was observed for $m/z > 95\,u$.

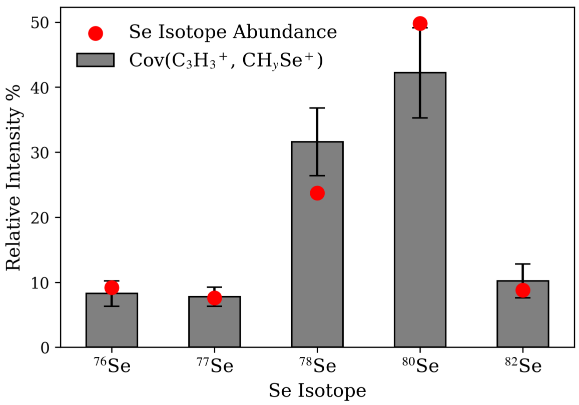

Figure S9: The summed relative intensities of fragmentation channels producing $C_3H_3^+$ and all CH_ySe^+ co-fragments incorporating the different selenium isotopes, also given in Table S9. These covariances made up $7.8 \pm 0.6\%$ of all covariances observed for the $C_nH_x{}^+ + C_{(4-n)}H_ySe^+$ (where $x + y \leq 4$) fragmentation channels. The error bars correspond to uncertainty values obtained from an adapted bootstrapping method outlined in the Methods section. Red dots indicate the natural selenium isotope abundances. The expected Se isotope abundances are also given by red dots.

CH_ySe^+ m/z	Intensity (arb. u.)	^{76}Se (9.2%)	^{77}Se (7.6%)	^{78}Se (23.7%)	^{80}Se (49.8%)	^{82}Se (8.8%)
88	0.5 ± 0.3	0.5 ± 0.3				
89	2.8 ± 0.7	1.5 ± 0.4	1.3 ± 0.3			
90	2.6 ± 0.7		0.6 ± 0.2	2.0 ± 0.5		
91	5.7 ± 1.1			5.7 ± 1.1		
92	1.4 ± 0.6				1.4 ± 0.6	
93	8.9 ± 1.6				8.9 ± 1.6	
94	0.6 ± 0.3					0.6 ± 0.3
95	1.9 ± 0.5					1.9 ± 0.5
	Intensity (%)	8.3 ± 1.9	7.8 ± 1.5	31.6 ± 5.2	42.2 ± 7.0	10.2 ± 2.6

Table S9: The integrated intensities of Newton frame covariance maps between $C_3H_3^+$ and CH_ySe^+ fragments ($m/z = 88 - 95\,u$). No covariance was observed for $m/z > 95\,u$.

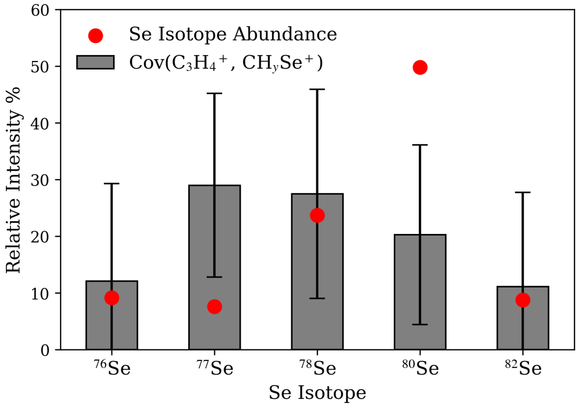

Figure S10: The summed relative intensities of fragmentation channels producing $C_3H_4{}^+$ and all CH_ySe^+ co-fragments incorporating the different selenium isotopes, also given in Table S10. These covariances made up $0.3 \pm 0.2\%$ of all covariances observed for the $C_nH_x{}^+ + C_{(4-n)}H_ySe^+$ (where $x + y \leq 4$) fragmentation channels. The error bars correspond to uncertainty values obtained from an adapted bootstrapping method outlined in the Methods section. Red dots indicate the natural selenium isotope abundances. The expected Se isotope abundances are also given by red dots.

CH_ySe^+ m/z	Intensity (arb. u.)	^{76}Se (9.2%)	^{77}Se (7.6%)	^{78}Se (23.7%)	^{80}Se (49.8%)	^{82}Se (8.8%)
88	0.2 ± 0.3	0.2 ± 0.3				
89	0.5 ± 0.3		0.5 ± 0.3			
90	0.5 ± 0.3			0.5 ± 0.3		
91	-					
92	0.4 ± 0.3				0.4 ± 0.3	
93	-					
94	0.2 ± 0.3					0.2 ± 0.3
95	-					
	Intensity (%)	12.1 ± 17.2	29.0 ± 16.2	27.5 ± 18.4	20.3 ± 15.8	11.1 ± 16.6

Table S10: The integrated intensities of Newton frame covariance maps between $C_3H_4{}^+$ and CH_ySe^+ fragments ($m/z = 88 - 95\,u$). No covariance was observed for $m/z > 95\,u$.

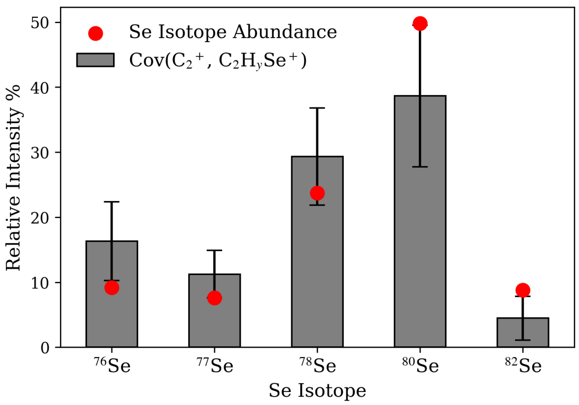

Figure S11: The summed relative intensities of fragmentation channels producing $C_2{}^+$ and all $C_2H_ySe^+$ co-fragments incorporating the different selenium isotopes, also given in Table S11. These covariances made up $1.5 \pm 0.2\%$ of all covariances observed for the $C_nH_x{}^+ + C_{(4-n)}H_ySe^+$ (where $x + y \leq 4$) fragmentation channels. The error bars correspond to uncertainty values obtained from an adapted bootstrapping method outlined in the Methods section. Red dots indicate the natural selenium isotope abundances. The expected Se isotope abundances are also given by red dots.

$C_2H_ySe^+$ m/z	Intensity (arb. u.)	^{76}Se (9.2%)	^{77}Se (7.6%)	^{78}Se (23.7%)	^{80}Se (49.8%)	^{82}Se (8.8%)
100	0.2 ± 0.2	0.2 ± 0.2				
101	0.4 ± 0.3	0.2 ± 0.2	0.2 ± 0.1			
102	0.5 ± 0.3	0.1 ± 0.1	0.1 ± 0.1	0.3 ± 0.2		
103	0.7 ± 0.3	0.2 ± 0.1	0.1 ± 0.1	0.4 ± 0.2		
104	0.8 ± 0.3		0.1 ± 0.03	0.2 ± 0.1	0.5 ± 0.2	
105	0.9 ± 0.4			0.3 ± 0.1	0.6 ± 0.3	
106	0.4 ± 0.2				0.3 ± 0.2	0.1 ± 0.04
107	0.2 ± 0.2				0.2 ± 0.2	0.03 ± 0.04
108	0.1 ± 0.1					0.1 ± 0.1
	Intensity (%)	16.3 ± 6.0	11.2 ± 3.7	29.3 ± 7.5	38.7 ± 10.9	4.5 ± 3.4

Table S11: The integrated intensities of Newton frame covariance maps between $C_2{}^+$ and $C_2H_ySe^+$ fragments ($m/z = 100 - 108\,u$).

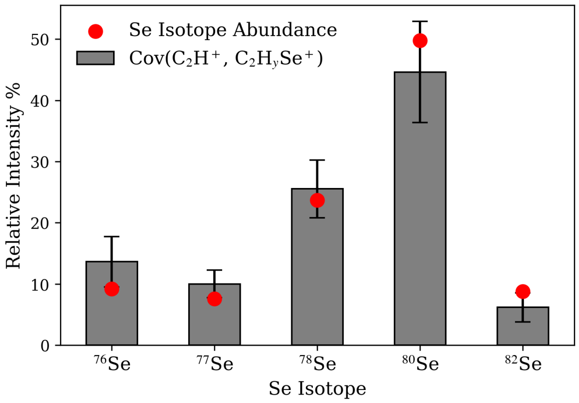

Figure S12: The summed relative intensities of fragmentation channels producing C_2H^+ and all $C_2H_ySe^+$ co-fragments incorporating the different selenium isotopes, also given in Table S12. These covariances made up $6.3 \pm 0.3\%$ of all covariances observed for the $C_nH_x{}^+ + C_{(4-n)}H_ySe^+$ (where $x + y \leq 4$) fragmentation channels. The error bars correspond to uncertainty values obtained from an adapted bootstrapping method outlined in the Methods section. Red dots indicate the natural selenium isotope abundances. The expected Se isotope abundances are also given by red dots.

$C_2H_ySe^+$ m/z	Intensity (arb. u.)	^{76}Se (9.2%)	^{77}Se (7.6%)	^{78}Se (23.7%)	^{80}Se (49.8%)	^{82}Se (8.8%)
100	0.4 ± 0.3	0.4 ± 0.3				
101	0.7 ± 0.4	0.4 ± 0.2	0.3 ± 0.2			
102	1.1 ± 0.5	0.2 ± 0.1	0.2 ± 0.1	0.6 ± 0.3		
103	1.3 ± 0.5	0.3 ± 0.1	0.3 ± 0.1	0.8 ± 0.3		
104	2.4 ± 0.8		0.2 ± 0.1	0.7 ± 0.2	1.5 ± 0.5	
105	1.3 ± 0.5			0.4 ± 0.2	0.9 ± 0.3	
106	1.6 ± 0.5				1.4 ± 0.5	0.2 ± 0.1
107	0.8 ± 0.4				0.7 ± 0.3	0.1 ± 0.1
108	0.2 ± 0.2					0.2 ± 0.2
	Intensity (%)	13.7 ± 4.1	10.0 ± 2.2	25.5 ± 4.7	44.6 ± 8.3	6.2 ± 2.4

Table S12: The integrated intensities of Newton frame covariance maps between C_2H^+ and $C_2H_ySe^+$ fragments ($m/z = 100 - 108\,u$).

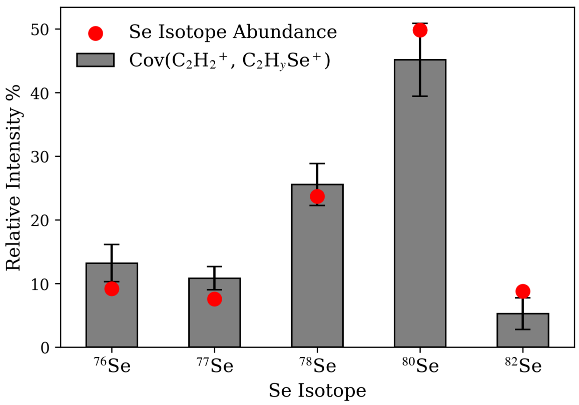

Figure S13: The summed relative intensities of fragmentation channels producing $C_2H_2^+$ and all $C_2H_ySe^+$ co-fragments incorporating the different selenium isotopes, also given in Table S13. These covariances made up $6.3 \pm 0.5\%$ of all covariances observed for the $C_nH_x^+ + C_{(4-n)}H_ySe^+$ (where $x + y \leq 4$) fragmentation channels. The error bars correspond to uncertainty values obtained from an adapted bootstrapping method outlined in the Methods section. Red dots indicate the natural selenium isotope abundances. The expected Se isotope abundances are also given by red dots.

$C_2H_ySe^+$ m/z	Intensity (arb. u.)	^{76}Se (9.2%)	^{77}Se (7.6%)	^{78}Se (23.7%)	^{80}Se (49.8%)	^{82}Se (8.8%)
100	0.8 ± 0.4	0.8 ± 0.4				
101	2.0 ± 0.7	1.1 ± 0.4	0.9 ± 0.3			
102	3.0 ± 0.7	0.7 ± 0.2	0.6 ± 0.1	1.7 ± 0.4		
103	2.8 ± 0.6		0.7 ± 0.1	2.1 ± 0.4		
104	3.7 ± 0.8			1.2 ± 0.3	2.5 ± 0.5	
105	4.9 ± 0.9				4.9 ± 0.9	
106	1.7 ± 0.6				1.5 ± 0.5	0.3 ± 0.1
107	0.5 ± 0.4					0.5 ± 0.4
108	0.3 ± 0.3					0.3 ± 0.3
	Intensity (%)	13.2 ± 2.9	10.8 ± 1.8	25.5 ± 3.3	45.2 ± 5.7	5.3 ± 2.5

Table S13: The integrated intensities of Newton frame covariance maps between $C_2H_2^+$ and $C_2H_ySe^+$ fragments ($m/z = 100 - 108\,u$).

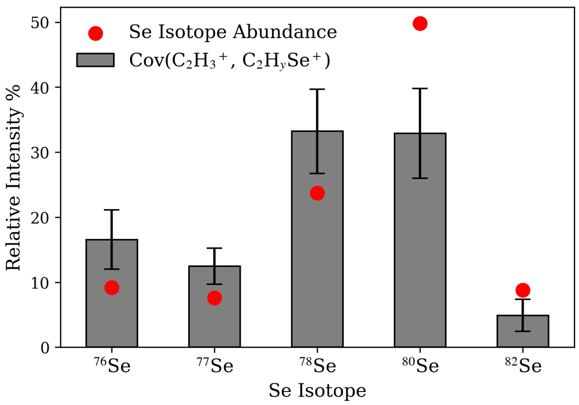

Figure S14: The summed relative intensities of fragmentation channels producing $C_2H_3^+$ and all $C_2H_ySe^+$ co-fragments incorporating the different selenium isotopes, also given in Table S14. These covariances made up $4.0 \pm 0.5\%$ of all covariances observed for the $C_nH_x^+ + C_{(4-n)}H_ySe^+$ (where $x + y \leq 4$) fragmentation channels. The error bars correspond to uncertainty values obtained from an adapted bootstrapping method outlined in the Methods section. Red dots indicate the natural selenium isotope abundances. The expected Se isotope abundances are also given by red dots.

$C_2H_ySe^+$ m/z	Intensity (arb. u.)	^{76}Se (9.2%)	^{77}Se (7.6%)	^{78}Se (23.7%)	^{80}Se (49.8%)	^{82}Se (8.8%)
100	1.1 ± 0.4	1.1 ± 0.4				
101	1.7 ± 0.7	1.0 ± 0.4	0.8 ± 0.3			
102	3.1 ± 0.6		0.8 ± 0.2	2.4 ± 0.5		
103	1.7 ± 0.6			1.7 ± 0.6		
104	2.9 ± 0.7				2.9 ± 0.7	
105	1.2 ± 0.4				1.2 ± 0.4	
106	0.5 ± 0.2					0.5 ± 0.2
107	0.1 ± 0.2					0.1 ± 0.2
	Intensity (%)	16.6 ± 4.6	12.5 ± 2.8	33.2 ± 6.5	32.9 ± 6.9	4.9 ± 2.5

Table S14: The integrated intensities of Newton frame covariance maps between $C_2H_3^+$ and $C_2H_ySe^+$ fragments ($m/z = 100 - 108\,u$).

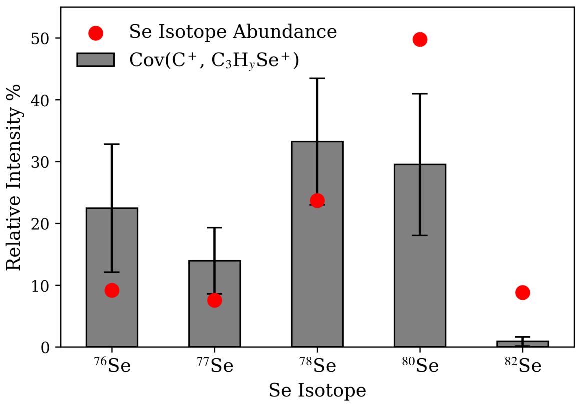

Figure S15: The summed relative intensities of fragmentation channels producing C^+ and all $C_3H_ySe^+$ co-fragments incorporating the different selenium isotopes, also given in Table S15. These covariances made up $1.1 \pm 0.2\%$ of all covariances observed for the $C_nH_x{}^+ + C_{(4-n)}H_ySe^+$ (where $x + y \leq 4$) fragmentation channels. The error bars correspond to uncertainty values obtained from an adapted bootstrapping method outlined in the Methods section. Red dots indicate the natural selenium isotope abundances. The expected Se isotope abundances are also given by red dots.

$C_3H_ySe^+$ m/z	Intensity (arb. u.)	^{76}Se (9.2%)	^{77}Se (7.6%)	^{78}Se (23.7%)	^{80}Se (49.8%)	^{82}Se (8.8%)
112	0.3 ± 0.3	0.3 ± 0.3				
113	0.3 ± 0.3	0.2 ± 0.2	0.1 ± 0.1			
114	0.6 ± 0.3	0.1 ± 0.1	0.1 ± 0.05	0.3 ± 0.2		
115	0.6 ± 0.4	0.1 ± 0.1	0.1 ± 0.1	0.4 ± 0.2		
116	0.8 ± 0.4		0.1 ± 0.04	0.2 ± 0.1	0.5 ± 0.3	
117	0.4 ± 0.3			0.1 ± 0.1	0.3 ± 0.2	
118	0.2 ± 0.2				0.2 ± 0.1	0.03 ± 0.02
	Intensity (%)	22.5 ± 10.4	13.9 ± 5.4	33.2 ± 10.2	29.5 ± 11.5	0.9 ± 0.7

Table S15: The integrated intensities of Newton frame covariance maps between C^+ and $C_3H_ySe^+$ fragments ($m/z = 112 - 118\,u$).

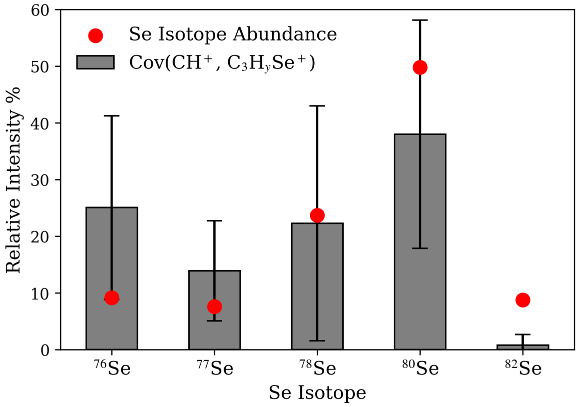

Figure S16: The summed relative intensities of fragmentation channels producing CH^+ and all $C_3H_ySe^+$ co-fragments incorporating the different selenium isotopes, also given in Table S16. These covariances made up $0.6 \pm 0.2\%$ of all covariances observed for the $C_nH_x{}^+ + C_{(4-n)}H_ySe^+$ (where $x + y \leq 4$) fragmentation channels. The error bars correspond to uncertainty values obtained from an adapted bootstrapping method outlined in the Methods section. Red dots indicate the natural selenium isotope abundances. The expected Se isotope abundances are also given by red dots.

$C_3H_ySe^+$ m/z	Intensity (arb. u.)	^{76}Se (9.2%)	^{77}Se (7.6%)	^{78}Se (23.7%)	^{80}Se (49.8%)	^{82}Se (8.8%)
112	0.2 ± 0.2	0.2 ± 0.2				
113	0.3 ± 0.2	0.2 ± 0.1	0.1 ± 0.1			
114	0.1 ± 0.3	0.01 ± 0.1	0.01 ± 0.1	0.03 ± 0.2		
115	0.1 ± 0.4	0.03 ± 0.1	0.02 ± 0.1	0.1 ± 0.2		
116	0.6 ± 0.3		0.1 ± 0.03	0.2 ± 0.1	0.4 ± 0.2	
117	0.3 ± 0.3			0.1 ± 0.1	0.2 ± 0.2	
118	0.1 ± 0.2				0.1 ± 0.2	0.01 ± 0.03
	Intensity (%)	25.0 ± 16.2	13.9 ± 8.8	22.3 ± 20.7	38.0 ± 20.1	0.8 ± 1.9

Table S16: The integrated intensities of Newton frame covariance maps between CH^+ and $C_3H_ySe^+$ fragments ($m/z = 112 - 118\,u$).

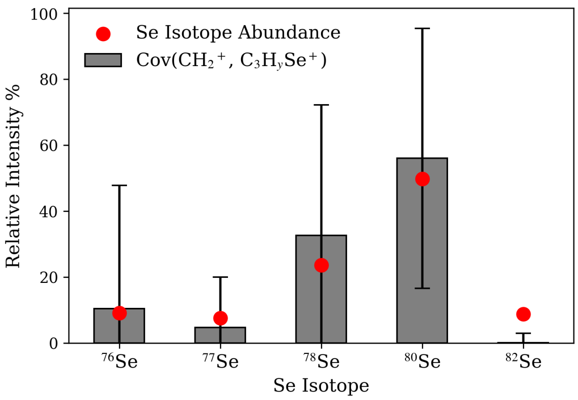

Figure S17: The summed relative intensities of fragmentation channels producing CH_2^+ and all $C_3H_ySe^+$ co-fragments incorporating the different selenium isotopes, also given in Table S17. These covariances made up $0.8 \pm 0.2\%$ of all covariances observed for the $C_nH_x{}^+ + C_{(4-n)}H_ySe^+$ (where $x + y \leq 4$) fragmentation channels. The error bars correspond to uncertainty values obtained from an adapted bootstrapping method outlined in the Methods section. Red dots indicate the natural selenium isotope abundances. The expected Se isotope abundances are also given by red dots.

$C_3H_ySe^+$ m/z	Intensity (arb. u.)	^{76}Se (9.2%)	^{77}Se (7.6%)	^{78}Se (23.7%)	^{80}Se (49.8%)	^{82}Se (8.8%)
112	0.04 ± 0.2	0.04 ± 0.2				
113	0.03 ± 0.2	0.02 ± 0.1	0.01 ± 0.1			
114	0.1 ± 0.4	0.01 ± 0.1	0.01 ± 0.1	0.03 ± 0.2		
115	0.1 ± 0.2		0.02 ± 0.1	0.1 ± 0.2		
116	0.5 ± 0.3			0.2 ± 0.1	0.4 ± 0.2	
117	0.1 ± 0.2				0.1 ± 0.2	
118	0.01 ± 0.2				0.01 ± 0.1	0.001 ± 0.02
	Intensity (%)	8.43 ± 29.02	4.7 ± 15.3	30.6 ± 36.0	56.0 ± 39.4	0.2 ± 2.8

Table S17: The integrated intensities of Newton frame covariance maps between CH_2^+ and $C_3H_ySe^+$ fragments ($m/z = 112 - 118\,u$).

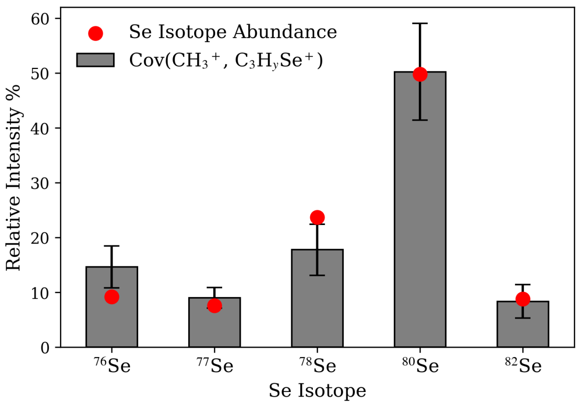

Figure S18: The summed relative intensities of fragmentation channels producing CH_3^+ and all $C_3H_ySe^+$ co-fragments incorporating the different selenium isotopes, also given in Table S18. These covariances made up $4.2 \pm 0.4\%$ of all covariances observed for the $C_nH_x^+ + C_{(4-n)}H_ySe^+$ (where $x + y \leq 4$) fragmentation channels. The error bars correspond to uncertainty values obtained from an adapted bootstrapping method outlined in the Methods section. Red dots indicate the natural selenium isotope abundances. The expected Se isotope abundances are also given by red dots.

$C_3H_ySe^+$ m/z	Intensity (arb. u.)	^{76}Se (9.2%)	^{77}Se (7.6%)	^{78}Se (23.7%)	^{80}Se (49.8%)	^{82}Se (8.8%)
112	1.6 ± 0.5	1.6 ± 0.5				
113	1.2 ± 0.5	0.7 ± 0.3	0.7 ± 0.2			
114	3.4 ± 0.8		0.8 ± 0.2	2.5 ± 0.6		
115	0.2 ± 0.4			0.2 ± 0.4		
116	7.5 ± 1.3				7.5 ± 1.3	
117	0.2 ± 0.3				0.2 ± 0.3	
118	1.3 ± 0.5					1.3 ± 0.5
	Intensity (%)	14.7 ± 3.8	9.0 ± 1.9	17.8 ± 4.7	50.2 ± 8.8	8.4 ± 3.0

Table S18: The integrated intensities of Newton frame covariance maps between CH_3^+ and $C_3H_ySe^+$ fragments ($m/z = 112 - 118\,u$).

2 All $C_nH_x^+/C_{(4-n)}H_ySe^+$ channel intensities

Table S19 lists the relative intensities of every observed reaction channel producing $C_nH_x^+$ and $C_{(4-n)}H_ySe^+$, with $n = 1 - 4$ and $x + y \leq 4$. This is a more comprehensive version of Table 2 from the main text and includes 22 additional channels with relative intensities that are each less than 1% of the total.

Product Pair	Fragmentation Channel	Relative Intensity (%)	Summed Intensity (%)
$C_4H_x{}^+ + SeH_y{}^+$	$C_4{}^+ + Se^+ + 4H^{0/+}$	1.6 ± 0.3	54.0 ± 1.7
	$C_4{}^+ + SeH^+ + 3H^{0/+}$	2.4 ± 0.4	
	$C_4{}^+ + SeH_2{}^+ + 2H^{0/+}$	1.0 ± 0.2	
	$C_4H^+ + Se^+ + 3H^{0/+}$	4.5 ± 0.4	
	$C_4H^+ + SeH^+ + 2H^{0/+}$	5.3 ± 0.6	
	$C_4H^+ + SeH_2{}^+ + H^{0/+}$	3.0 ± 0.3	
	$C_4H_2{}^+ + Se^+ + 2H^{0/+}$	7.9 ± 0.7	
	$C_4H_2{}^+ + SeH^+ + H^{0/+}$	7.9 ± 0.6	
	(i) $C_4H_2{}^+ + SeH_2{}^+$	5.6 ± 0.5	
	$C_4H_3{}^+ + Se^+ + H^{0/+}$	6.1 ± 0.6	
	(ii) $C_4H_3{}^+ + SeH^+$	6.1 ± 0.6	
	(iii) $C_4H_4{}^+ + Se^+$	2.6 ± 0.4	
$C_3H_x{}^+ + CH_ySe^+$	$C_3{}^+ + CSe^+ + 4H^{0/+}$	0.7 ± 0.2	23.2 ± 1.1
	$C_3{}^+ + CHSe^+ + 3H^{0/+}$	1.0 ± 0.2	
	$C_3{}^+ + CH_2Se^+ + 2H^{0/+}$	0.8 ± 0.2	
	$C_3{}^+ + CH_3Se^+ + H^{0/+}$	1.1 ± 0.3	
	$C_3H^+ + CSe^+ + 3H^{0/+}$	1.1 ± 0.2	
	$C_3H^+ + CHSe^+ + 2H^{0/+}$	1.6 ± 0.3	
	$C_3H^+ + CH_2Se^+ + H^{0/+}$	1.0 ± 0.2	
	(iv) $C_3H^+ + CH_3Se^+$	1.6 ± 0.3	
	$C_3H_2{}^+ + CSe^+ + 2H^{0/+}$	1.3 ± 0.3	
	$C_3H_2{}^+ + CHSe^+ + H^{0/+}$	3.4 ± 0.4	
	$C_3H_2{}^+ + CH_2Se^+$	0.9 ± 0.2	
	$C_3H_3{}^+ + CSe^+ + H^{0/+}$	1.9 ± 0.3	
	(v) $C_3H_3{}^+ + CHSe^+$	6.3 ± 0.7	
	$C_3H_4{}^+ + CSe^+$	0.6 ± 0.2	
$C_2H_x{}^+ + C_2H_ySe^+$	$C_2{}^+ + C_2Se^+ + 4H^{0/+}$	0.4 ± 0.1	15.7 ± 0.8
	$C_2{}^+ + C_2HSe^+ + 3H^{0/+}$	0.5 ± 0.1	
	$C_2{}^+ + C_2H_2Se^+ + 2H^{0/+}$	0.3 ± 0.1	
	$C_2{}^+ + C_2H_3Se^+ + H^{0/+}$	0.2 ± 0.1	
	$C_2H^+ + C_2Se^+ + 3H^{0/+}$	1.0 ± 0.2	
	$C_2H^+ + C_2HSe^+ + 2H^{0/+}$	0.8 ± 0.2	
	$C_2H^+ + C_2H_2Se^+ + H^{0/+}$	1.0 ± 0.2	
	$C_2H^+ + C_2H_3Se^+$	0.6 ± 0.1	
	$C_2H_2{}^+ + C_2Se^+ + 2H^{0/+}$	2.1 ± 0.3	
	$C_2H_2{}^+ + C_2HSe^+ + H^{0/+}$	3.1 ± 0.4	
	(vi) $C_2H_2{}^+ + C_2H_2Se^+$	1.5 ± 0.2	
	$C_2H_3{}^+ + C_2Se^+ + H^{0/+}$	2.6 ± 0.4	
	(vii) $C_2H_3{}^+ + C_2HSe^+$	1.6 ± 0.3	
$CH_x{}^+ + C_3H_ySe^+$	$C^+ + C_3Se^+ + 4H^{0/+}$	0.4 ± 0.1	7.1 ± 0.7
	$C^+ + C_3HSe^+ + 3H^{0/+}$	0.3 ± 0.1	
	$C^+ + C_3H_2Se^+ + 2H^{0/+}$	0.2 ± 0.1	
	$C^+ + C_3H_3Se^+ + H^{0/+}$	0.1 ± 0.05	
	$CH^+ + C_3Se^+ + 3H^{0/+}$	0.2 ± 0.1	
	$CH^+ + C_3HSe^+ + 2H^{0/+}$	0.1 ± 0.1	
	$CH^+ + C_3H_2Se^+ + H^{0/+}$	0.1 ± 0.1	
	$CH^+ + C_3H_3Se^+ +$	0.1 ± 0.04	
	$CH_2{}^+ + C_3Se^+ + 2H^{0/+}$	0.2 ± 0.1	
	$CH_2{}^+ + C_3HSe^+ + H^{0/+}$	0.1 ± 0.1	
	$CH_2{}^+ + C_3H_2Se^+$	0.1 ± 0.1	
	$CH_3{}^+ + C_3Se^+ + H^{0/+}$	4.5 ± 0.6	
	(viii) $CH_3{}^+ + C_3HSe^+$	0.6 ± 0.2	
	Total	100 ± 2.3	

Table S19: The relative integrated intensities of 'two-body' fragmentation channels obtained via Newton-frame covariance, bootstrapping analysis and isotope abundance considerations.

3 $H^{0/+}$ dynamics in channels producing $C_nH_x^+$ and $C_{(4-n)}H_ySe^+$

Table S19 indicates that 72% of the 'two-body' $C_nH_x^+/C_{(4-n)}H_ySe^+$ channels lose at least one $H^{0/+}$ species. More instructive information about these $H^{0/+}$ dynamics can be gained from the corresponding fragment momentum distributions provided by Newton-frame covariance imaging. The $C_nH_x^+/C_{(4-n)}H_ySe^+$ covariances can be grouped into two distinct categories that inform on the behaviour of the $H^{0/+}$ species: first, those where hydrogen atoms are missing (i.e., where $x + y < 4$); and second, those that exhibit hydrogen migration (e.g., as in the C_3H^+/CH_3Se^+ case, where the selenium-containing fragment has more hydrogens than would be expected from the parent structure).

Figures S19(a) and (b) illustrate how the summed kinetic energy (ΣKE) of the $C_nH_x^+/C_{(4-n)}H_ySe^+$ products in these categories varies. To focus the discussion, we have only included covariances involving fragments that contain the most abundant selenium isotope, ^{80}Se, as these yield the most intense and well-resolved features. However, as discussed in the main text, it should be noted that these may include contributions from other, more minor, fragmentation channels that generate products with the same m/z, which adds some uncertainty to the summed kinetic energies. A large kinetic energy spread is seen for both categories. As such, subsets of the data were considered in order to independently assess the effects of $H^{0/+}$ loss or migration. These are shown in Figures S19(c) and (d), respectively. The former demonstrates that, for the 18 channels observed to lose hydrogen atoms without any clear evidence of migration, there is a slight increase in kinetic energy once three or more hydrogens are lost, suggesting that most of these are neutral. This is consistent with the formation of parent di- or trications, as discussed in the main text. Figure S19(d) illustrates that, in the 7 cases where no hydrogens are lost but some migration occurs, the kinetic energy significantly decreases when at least two hydrogens are rearranged. This indicates a larger intercharge separation distance, and potentially a more extended (possibly ring-opened) structure prior to the fragmentation.

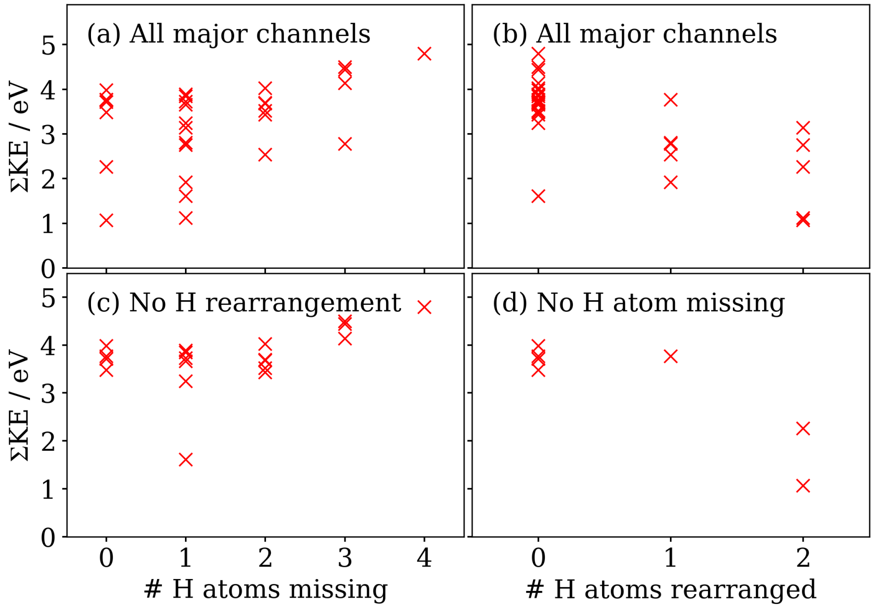

Figure S19: The summed kinetic energies (ΣKE) of carbon-containing products of $C_nH_x{}^+/C_{(4-n)}H_ySe^+$ channels involving the ^{80}Se isotope, plotted as a function of (a) the number of hydrogen atoms lost, and (b) the number of hydrogen atoms that migrate between fragments. Panels (c) and (d) respectively present subsets of the data in (a) and (b) where no hydrogen migration or loss has occurred.

4 Many-body selenophene fragmentation

The approximately back-to-back (180°) recoil exhibited by the covariant fragments in Figure 3 suggests they are the only heavy ions involved in their reaction channels, as the presence of a third heavy ion would perturb their trajectories by electrostatic repulsion. As such, their production must be accompanied by a neutral carbon-containing fragment.

To identify the mechanism, it is useful to consider whether the fragment momenta are indicative of a concerted or sequential process [1]. Consider a generic triatomic dication, ABC^{2+}, that fragments concertedly or sequentially as follows:

$$ABC^{2+} \rightarrow A^{+} + B^{+} + C \tag{4}$$

$$ABC^{2+} \rightarrow A^{+} + BC^{+} \rightarrow A^{+} + B^{+} + C \tag{5}$$

$$ABC^{2+} \rightarrow AB^{2+} + C \rightarrow A^{+} + B^{+} + C \tag{6}$$

If the fragmentation is concerted, as in reaction (4), it will primarily be driven by Coulombic repulsion, such that the primary momenta ($\vec{p}_{\mathrm{I}}$) of the ionic fragments will essentially be equal and opposite, with the neutral receiving very little impulse. However, if the molecule fragments through an initial charge separation step followed by secondary neutral dissociation, as in reaction (5), the subsequent dissociation of the intermediate product into neutral and ionic species partitions the primary momentum gained from the Coulombic repulsion into the secondary products according to their masses. The secondary products also gain an additional momentum component, $\vec{p}_{\mathrm{II}}$, from the neutral dissociation. In this case, the momentum of the secondary ion can be expressed as [1]:

$$\vec{p}_{\mathrm{B}} = -\frac{m_{\mathrm{B}}\vec{p}_{\mathrm{I}}}{m_{\mathrm{BC}}} + p_{\mathrm{II}}. \tag{7}$$

Finally, if the molecule instead fragments through deferred charge separation, as in reaction (6), the corresponding momentum of the secondary ion will be as follows (where $\vec{p}_{\mathrm{I}}$ and $\vec{p}_{\mathrm{II}}$ now represent neutral dissociation and charge separation, respectively):

$$\vec{p}_{\mathrm{B}} = -\frac{m_{\mathrm{B}}\vec{p}_{\mathrm{I}}}{m_{\mathrm{AB}}} + p_{\mathrm{II}}. \tag{8}$$

Since $\vec{p}_{\mathrm{II}}$ is much less than $\vec{p}_{\mathrm{I}}$ in equation (7), but much greater than $\vec{p}_{\mathrm{I}}$ in equation (8), it can be seen that the momentum of the secondary ion is approximately the same for reactions (4) and (6), but different for reaction (5), which enables the latter to be isolated. This was carried out by restricting the Newton-frame covariance analysis to only include fragments whose momenta satisfied equation (7). The value of $\vec{p}_{\mathrm{II}}$ was set to 30 a.u., which provided some leeway to account for the small amount of momentum gained by the neutral dissociation, as well as additional contributions from potential $H^{0/+}$-loss processes (see Section 6 for further details). This value was empirically determined to produce the best contrast between the fragmentation channels shown in Figure 3.

5 Probe pulse energy distribution

Figure S20 displays the attenuated SACLA pulse energy distribution within the interaction region of the spectrometer during the reported experiments. Its Gaussian mean and standard deviation are $1.9 \pm 0.2\,\mu$J. This data was subsequently divided into ten bins with equal numbers of laser shots in order to perform contingent covariance analysis, as described in the main text [2, 3]. The bounds of these subsets are shown by the dashed lines.

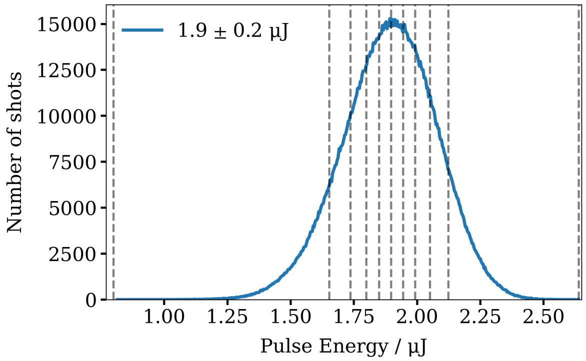

Figure S20: A histogram of the attenuated 120 eV pulse energies. The Gaussian mean and standard deviation are $1.9 \pm 0.2\,\mu$J. The vertical lines indicate the boundaries of ten bins containing equal numbers of laser shots, which were used for contingent covariance analysis.

6 Covariance analysis constraints

In the present experiment, velocity-map imaging produces Newton spheres of ions that, for closely-spaced m/z peaks, can remain partially overlapped as they reach the detector. This results in broad structures along the time-of-flight (m/z) coordinate with unclear boundaries for the starting and ending points of the Newton spheres involved. For example, the m/z signals in Figures S21(a) and S21(b) respectively contain overlapping contributions from $C_4H_x^+$ ($x = 0 - 4$) and Se^+/SeH_y^+ (with all Se isotopes, and $y = 1 - 2$). Selecting a wide time-of-flight range (indicated by pairs of vertical lines) for their Newton-frame covariance analysis leads to the appearance of non-physical features in the resulting covariance map, which can be attributed to the inclusion of ions from other, uncorrelated Newton spheres. For example, Figures S21(c)-(f) display the Newton-frame covariance maps of $^{80}Se^+/^{78}SeH_2^+$ with respect to $C_4H_2^+$ for different time-of-flight ranges (in nanoseconds). These ions originate from two-body processes (ignoring $H^{0/+}$ loss) and should therefore exhibit back-to-back recoil. However, shallower recoil angles are also observed in (c)-(e). These latter features are non-physical and disappear when narrower time-of-flight ranges are used for the analysis. These constraints effectively restrict the data used for the covariance calculations to ions that are within the central slices of the Newton spheres; that is, those with momenta distributed perpendicular to the time-of-flight axis. Figure S21(f) demonstrates that these limits virtually eliminate the appearance of the non-physical features, but retain the signal of interest. A time-of-flight window of ± 10 ns provides an optimal balance that reduces Newton-sphere overlap whilst retaining as much signal as possible from the feature of interest. We note that, since time-of-flight is non-linearly related to m/z, this filter selects different m/z fractions per ion species. Choosing the same, still narrow, m/z fraction for each ion species instead does not significantly change the extracted relative intensities of covariances. Furthermore, filtering the data using the same m/z does not account for the various contributions of different fragmentation channels that generate products with different momenta and dispersion within the respective Newton sphere. Therefore, uniform time-of-flight limits were chosen across all ion species for this analysis.

A second constraint was applied to the Newton-frame covariance calculations to improve the signal-to-noise ratio when integrating the intensities of covariance maps. Fragments generated from two-body breakup processes have equal and opposite momenta. Their total three-dimensional momentum, as determined by the measured $\vec{p}_x$, $\vec{p}_y$ and $\vec{p}_z$ components, should therefore sum to zero. However, $H^{0/+}$ loss imparts a small amount of momentum to these fragments and hence changes this sum. To account for this effect, a total momentum limit, p_{lim}, was applied to the covariance calculations, which ensured that the three-dimensional momentum components of the ion pairs (A and B) obeyed the following equation:

$$\vec{p}_{\text{x,y,z}}(\text{A}) + \vec{p}_{\text{x,y,z}}(\text{B}) = 0 \pm p_{\text{lim}}. \tag{9}$$

The above equation also accounts for the inherent momentum resolution of the VMI spectrometer. This was approximately eight atomic units (a.u.) under the applied experimental conditions [4], meaning that even the measured momenta of ions generated from purely two-body mechanisms may not always sum to zero.

Figure S22 illustrates the effects of setting p_{lim} at 100 a.u. and 20 a.u. for two ion pairs that differ by their signal-to-noise ratios. In the first, which examines the momentum distribution of CH_ySe^+ ($m/z = 93\,u$) with respect to $C_3H_3{}^+$ (i.e., the example used in Figure 2 of the main text), the covariant feature is not visibly altered when the momentum limit is decreased. In the second, an ion pair with a much lower signal-to-noise ratio is considered. Here, the momentum distribution of CH_ySe^+ is plotted with respect to C_3H^+, which is less abundant than $C_3H_3{}^+$. In this case, background noise with comparable intensity to the feature of interest is observed when p_{lim} is set at 100 a.u., making it difficult to isolate the feature of interest. By contrast, this noise is mostly removed when the limit is lowered. Further reducing p_{lim} visibly depletes the features of interest, as this begins to approach the momentum resolution of the spectrometer. A p_{lim} of 20 a.u. was therefore used in all $C_nH_x{}^+/C_{(4-n)}H_ySe^+$ covariance calculations to improve their signal-to-noise ratios.

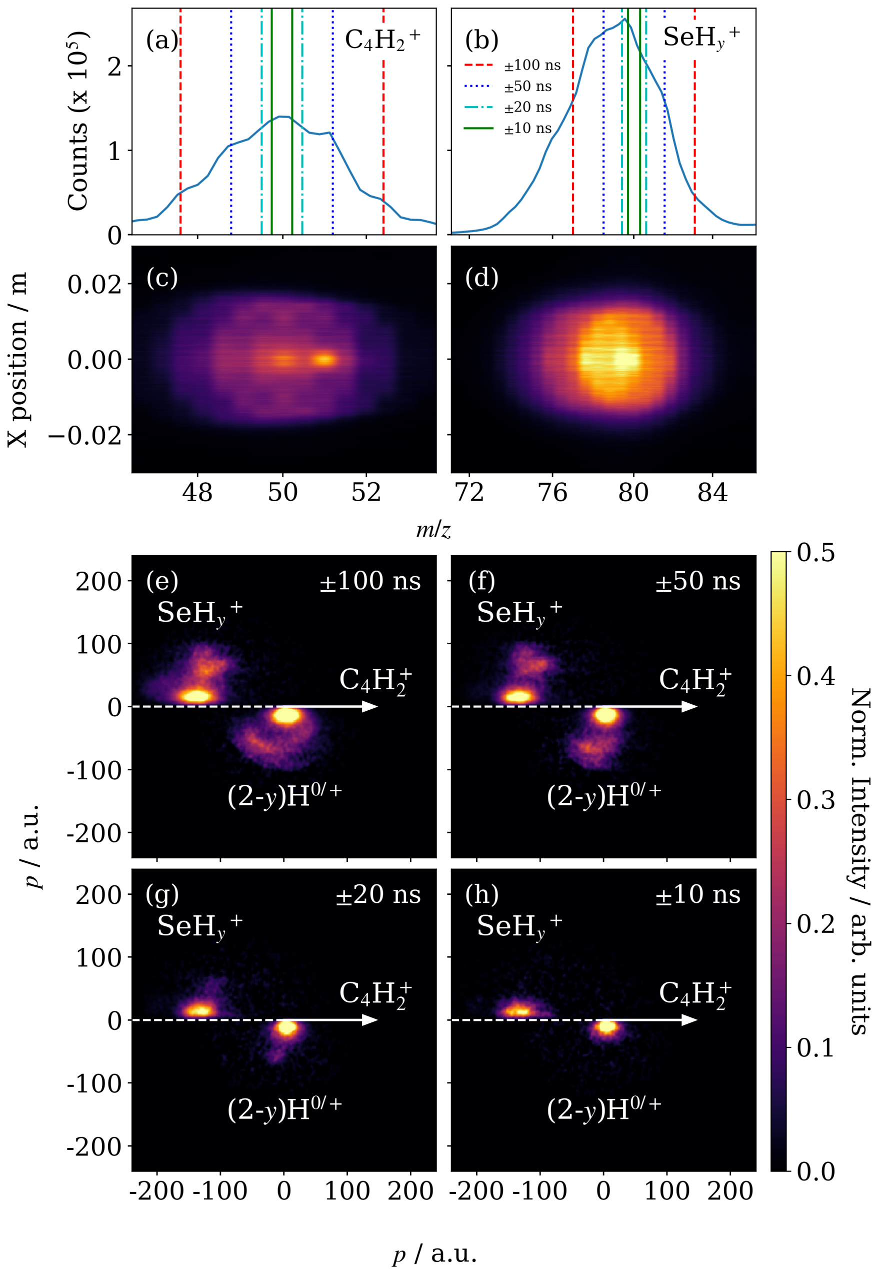

Figure S21: (a) and (b) show the m/z spectra for $C_4H_x{}^+$ and $SeH_y{}^+$ fragments, respectively; (c) and (d) show the m/z spectra for both fragments with respect to the ion hit position on the detector. The regions indicated by pairs of dashed lines in (a) and (b) were used to produce the Newton-frame covariance maps in (e)-(h), which show the recoil of $SeH_y{}^+$ ($m/z = 80\,u$) ions relative to $C_4H_2{}^+$ ions. Non-physical, off-180° features are observed in (e)-(g) due to contributions from neighboring fragments whose Newton spheres overlap with the ions of interest. The narrowest highlighted regions in (a) and (b) were used to calculate the Newton-frame covariance map (h), where the appearance of the non-physical features seen in (e)-(g) have been reduced. Each Newton-frame covariance map is normalized to its own maximum, with a color scale set to 0.5 arb. u. to better visualize the non-physical features.

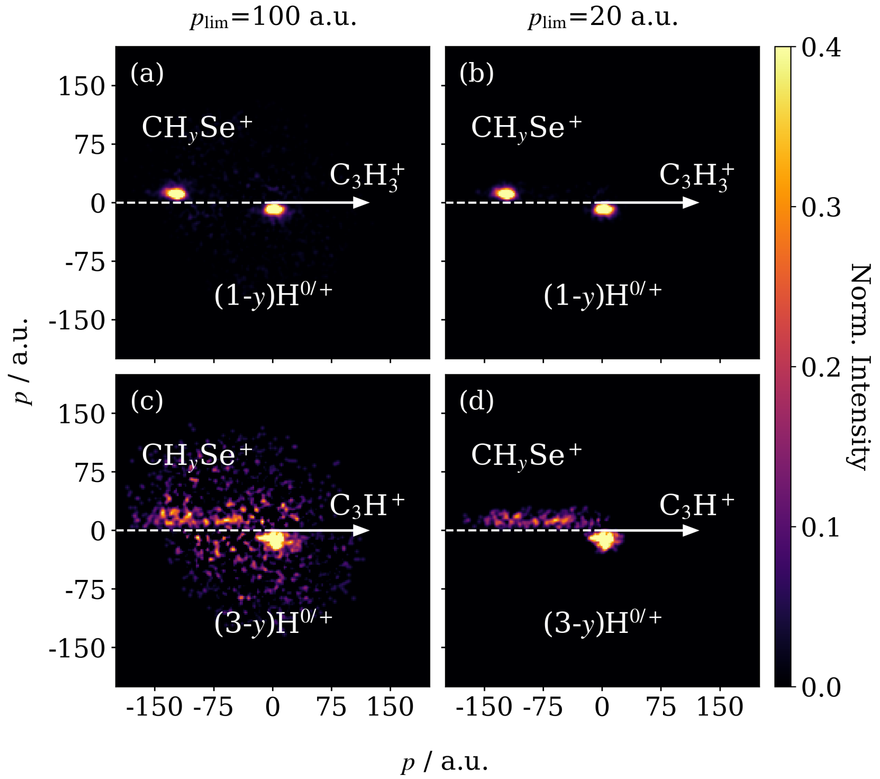

Figure S22: Newton-frame covariance maps of the momenta of CH_ySe^+ ions ($m/z = 93\,u$) relative to $C_3H_3{}^+$ calculated with a $p_{\rm lim}$ of (a) 100 a.u. and (b) 20 a.u.; (c) and (d) show the momenta of CH_ySe^+ relative to C_3H^+ ions with the same $p_{\rm lim}$ values as (a) and (b), respectively. Each covariance map is normalized to its own maximum, with the image contrast set to 0.4 arb. u. to best visualize the effect of varying $p_{\rm lim}$ on background noise reduction.

References

[1] J. H. D. Eland, "The dynamics of three-body dissociations of dications studied by the triple coincidence technique PEPIPICO," *Mol. Phys.*, vol. 61, no. 3, pp. 725–745, 1987.

[2] V. Zhaunerchyk, L. J. Frasinski, J. H. D. Eland, and R. Feifel, "Theory and simulations of covariance mapping in multiple dimensions for data analysis in high-event-rate experiments," *Phys. Rev. A*, vol. 89, no. 5, p. 053418, 2014.

[3] J. W. McManus, T. Walmsley, K. Nagaya, J. R. Harries, Y. Kumagai, H. Iwayama, M. N. Ashfold, M. Britton, P. H. Bucksbaum, B. Downes-Ward, *et al.*, "Disentangling sequential and concerted fragmentations of molecular polycations with covariant native frame analysis," *Phys. Chem. Chem. Phys.*, vol. 24, no. 37, pp. 22699–22709, 2022.

[4] T. Walmsley, J. W. McManus, Y. Kumagai, K. Nagaya, J. Harries, H. Iwayama, M. N. Ashfold, M. Britton, P. H. Bucksbaum, B. Downes-Ward, *et al.*, "The role of momentum partitioning in covariance ion imaging analysis," *J. Phys. Chem. A*, vol. 128, no. 22, pp. 4548–4560, 2024.
